# Hydrogen bonding triggered tunable high temperature phosphorescence

**DOI:** 10.1039/d6sc03982k

**Published:** 2026-08-05

**Authors:** Sheng-Qi Qiu, Jun-Ran Chen, Yao Xiao, Ming-Zhu Liao, Anqi He, Cai-Zhen Zhu, Ben Zhong Tang, Zhen-Qiang Yu

**Affiliations:** a College of Chemistry and Environmental Engineering, Shenzhen University Shenzhen 518071 China zqyu@szu.edu.cn czzhu@szu.edu.cn; b Beijing National Laboratory for Molecular Sciences, College of Chemistry and Molecular Engineering, Peking University Beijing 100871 P. R. China; c Guangdong Basic Research Center of Excellence for Aggregate Science, School of Science and Engineering, The Chinese University of Hong Kong (Shenzhen) Longgang Shenzhen Guangdong 518172 China tangbenz@cuhk.edu.cn

## Abstract

Due to the luminescence quenching at elevated temperature, the development of high temperature phosphorescent (HTP) materials with tunable emission remains a formidable challenge. Herein, a hydrogen bonding (HB) triggered strategy to achieve color-tunable and thermally robust room temperature phosphorescence (RTP) was demonstrated. Using a triazine derivative (TRZ) as a luminescent core and HB acceptor and a series of acids, *o*-pyridinesulfonic acid (OPS), ethanesulfonic acid (ESA), or trifluoroacetic acid (TFA), as HB donors, a series of stable HB luminescent systems were constructed, which not only exhibited RTP activity, but also enabled continuous wavelength tuning from 528 to 585 nm through varying acid strength. Remarkably, these HB systems exhibited phosphorescence that persisted up to 463 K, owing to the exceptional thermal stability, which was one of the highest reported working temperatures for tunable organic phosphors. By doping TRZ and ESA into a PMMA matrix, the material reveals promoted aggregation and stabilized triplet excitons, which lead to photoactivated RTP and a pronounced photothermal effect. Results also reveal that HB enhances spin–orbit coupling, promotes charge-transfer character, and restricts molecular motions, thereby suppressing thermal quenching. This work establishes a versatile strategy for designing tunable HTP materials with secondary interaction, broadening avenues for applications in high temperature optoelectronics and sensing.

## Abstract 2

Hydrogen bonding between a triazine derivative and acids of different strengths enables tunable phosphorescence from 528 to 585 nm that persists up to 463 K, offering a versatile design for tunable high temperature phosphorescent materials.

## Graphical abstract

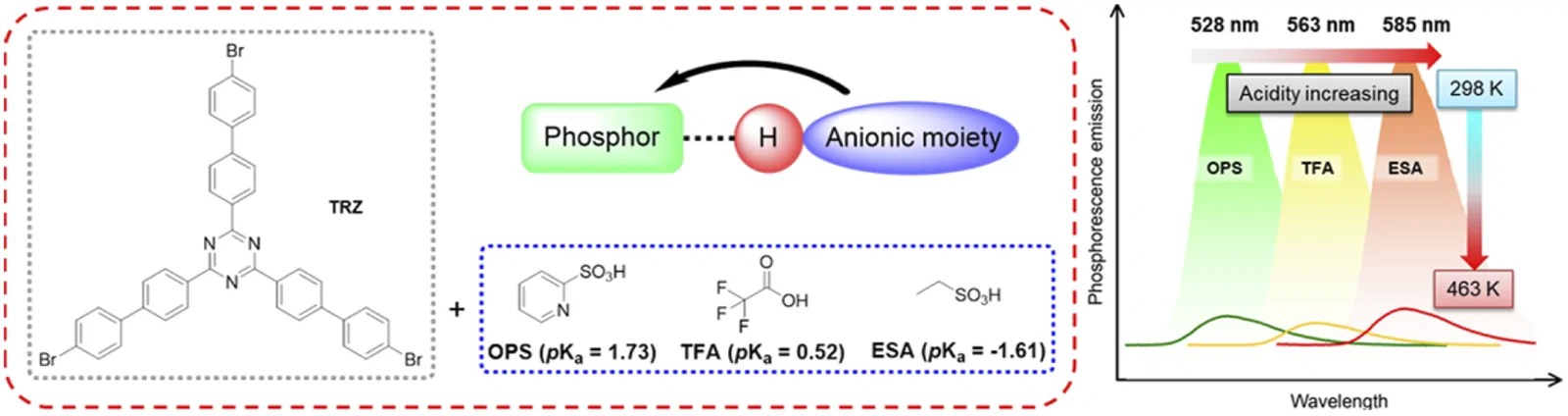

## Introduction

Phosphorescence is a radiative transition process originating from the triplet excited state to the ground state of a molecule. However, the excited triplet states of conventional phosphors, especially pure organic phosphors, are highly susceptible to non-radiative decay induced by various environmental factors, thus leading to phosphorescence quenching. Among these factors, temperature plays a particularly critical role. In general, elevated temperatures intensify molecular vibrations and rotations, significantly increasing the rate of non-radiative transitions and causing a dramatic decrease in phosphorescence intensity.^1–3^ With the advancement of modern technology into extreme environments, such as high-power lighting and displays^4^ and internal monitoring of engines,^5^ a growing demand for optical materials that can be stably operated at high temperature is rising. In such extreme environments, high temperature phosphorescence (HTP) enables critical non-contact temperature mapping and stable light emission. Therefore, the development of HTP materials has emerged as an important research direction.^6–9^ Beyond achieving stable HTP, regulating the emission wavelength would significantly broaden the application scope of phosphorescent materials. Despite the clear need, systematic studies directly addressing the tunability of phosphorescence at elevated temperatures remain scarce.^10–13^

To achieve tunable phosphorescence properties, researchers have focused considerable attention on stimuli-responsive room temperature phosphorescence (RTP) materials. Various regulation strategies, including solvent processing,^14,15^ phase modulation,^16–18^ acid responsiveness,^19–21^ and doping within host matrices,^22–24^ have been extensively developed over the past decade. As a classic and well-established strategy in modulating singlet luminophores, acid stimulation stands out as a promising approach for switching phosphorescence behavior among these strategies.^25–29^ The widely accepted mechanism for acid-responsive RTP involves the protonation of the phosphor, which alters its electronic structure.^29^ Nevertheless, the protonation mechanism requires strong basic functional groups, such as pyridine or tertiary amines, significantly restricting the scope of molecular design.^28,30,31^ In contrast, hydrogen bonding (HB) offers an alternative pathway that does not rely on protonation. However, existing HB-based RTP systems have been predominantly demonstrated under weak acid or neutral conditions,^9,11,32^ while the strong acid case, where protonation is conventionally expected, remains largely unexplored. Consequently, a critical question arises, for weak basic groups that cannot be protonated, what mechanism directed the acid response of RTP performance, especially when encountering strong acids? Weak basic groups usually possess lone pair electrons, which can engage in hydrogen bonding (HB) with acids, offering a distinct pathway for RTP control. Unlike protonation, which solely relies on the proton to switch RTP on/off, HB engages the entire acid molecule, allowing its anionic moiety to directly modulate triplet energy levels through non-covalent interactions. This enables not only triggering RTP but also the precise tuning of emission properties (Fig. 1a, left). Furthermore, HB has been proven to be an effective strategy to inhibit the non-radiative transition of phosphors,^9,11,32^ offering a promising route to tunable HTP (Fig. 1a, right). In this work, we demonstrate that through rational phosphor molecular design, even with strong acids (p*K*_a_ < 0), a stable HB complex can form instead of a protonated product, thereby extending the HB strategy into the strong-acid territory and achieving continuously tunable high-temperature phosphorescence.

**Fig. 1:**
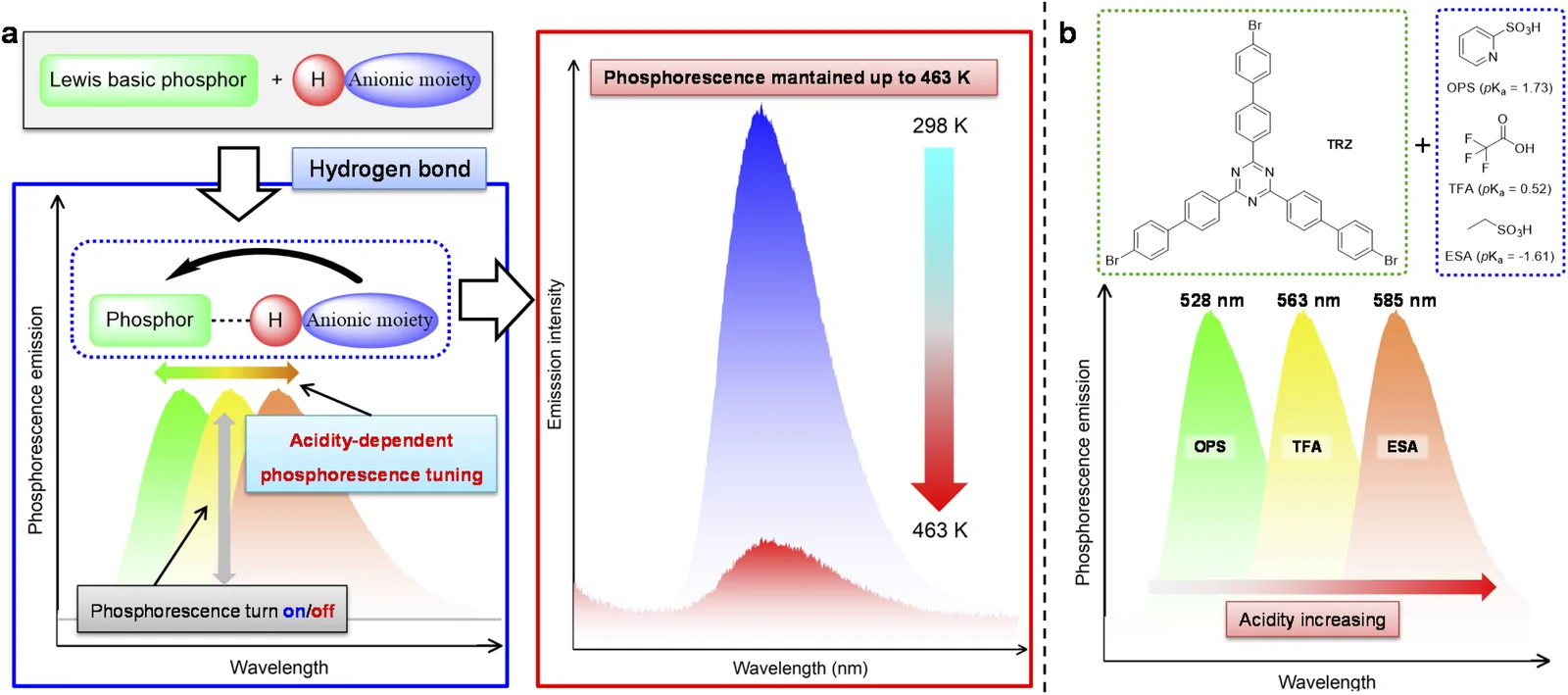
Acidity-dependent tunable high-temperature phosphorescence. (a) HB mechanism in acid-regulated phosphorescence and the high temperature resistance. (b) Tunable phosphorescence of TRZ tuned using acids.

In our previous work, a triazine derivative, TRZ, demonstrated photo-thermo-triggered RTP and acid-responsive RTP gating.^33,34^ In this study, confirmatory experiments attribute the acid-responsive properties to HB between TRZ and a series of acid molecules rather than to a protonation mechanism, even in the presence of a strong acid with p*K*_a_ < 0. Furthermore, the HB not only triggered phosphorescence but also enabled acidity-dependent control over the emission wavelength (*λ*), lifetime (*τ*), and quantum yield (*Φ*). The HB system in the solid state exhibited remarkable stability, was mechanically robust and independent of crystallization, and could even withstand high temperatures up to 463 K while retaining phosphorescence. Doping TRZ and ethanesulfonic acid (ESA) into a polymethyl methacrylate (PMMA) matrix further demonstrated that HB promotes aggregation and also stabilizes triplet excitons, resulting in photoactivated RTP and a pronounced photothermal effect with a temperature of up to 419 K within 12 s.

## Results and discussion

After synthesizing TRZ according to the procedure reported in the literature, the chemical structure of TRZ was confirmed by comparing with the literature.^33^ The photophysical behaviors were investigated by UV-Vis absorption, steady-state photoluminescence (PL) spectra, and time-resolved PL spectroscopy. The frontier molecular orbital profiles and spin–orbit coupling (SOC) of TRZ were calculated using density functional theory (DFT) at the B3LYP-D3(BJ)/6-31G(d,p) level of theory (for details see the SI).

The absorption (Fig. 2a and c) and PL spectra (Fig. 2b and d) of TRZ were measured in DCM solutions after being filtered through PTFE filter membranes with 0.22 µm pore size to avoid the interference from aggregation (Fig. S1). A single absorption peak at 318 nm was observed in TRZ solution at low concentration (Fig. 2a, black dashed line, 1.0 × 10^−5^ mol L^−1^). Adding 1 equivalent of ESA with the same concentration caused no effect on absorption (Fig. 2a, blue dashed line, 1.0 × 10^−5^ mol L^−1^). When the solution concentration was increased to 1.0 × 10^−3^ mol L^−1^ (Fig. 2a, black solid line), the sharp absorption was red-shifted to 353 nm, which was attributed to the enhanced intermolecular interaction in the concentrated solution. With the addition of ESA into the solution of 1.0 × 10^−3^ mol L^−1^, a broad absorption peak attributed to charge transfer (CT) was observed in the region from 390 to 500 nm (Fig. 2a, red solid line). In the solution of 1.0 × 10^−5^ mol L^−1^, the emission of TRZ peaked at 403 nm (Fig. 2b, black), slightly red-shifted to 412 nm (Fig. 2b, blue) with the addition of ESA under 318 nm excitation, and further red-shifted to 529 nm (Fig. 2b, red) as the concentration increased to 1.0 × 10^−3^ mol L^−1^. For the DCM solution without ESA, even when the concentration was increased to 1.0 × 10^−3^ mol L^−1^, the emission peak remained nearly unchanged (Fig. S2).

**Fig. 2:**
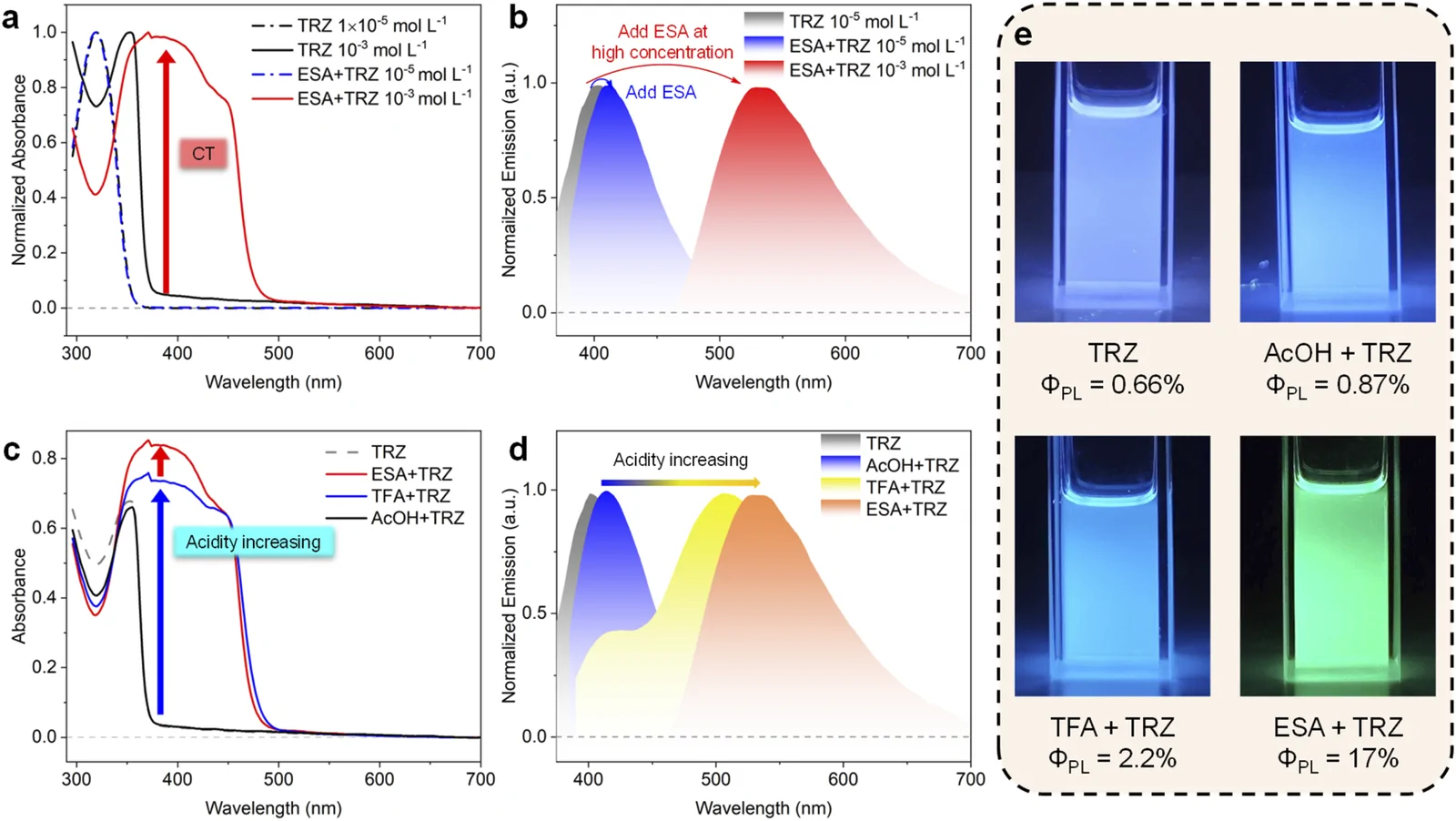
Photophysical properties of TRZ in DCM solutions. (a) Normalized absorption of TRZ and TRZ-ESA (1 : 1 in mole ratio) in DCM solution at 1.0 × 10^−3^ mol L^−1^ and 1.0 × 10^−5^ mol L^−1^. (b) Normalized emission of TRZ and TRZ-ESA (1 : 1 in mole ratio) in DCM solution at 1.0 × 10^−3^ mol L^−1^ and 1.0 × 10^−5^ mol L^−1^ under 318 nm UV excitation. (c) Absorption of TRZ with different acids in DCM solution at 1.0 × 10^−3^ mol L^−1^ concentration. (d) Normalized emission of TRZ and TRZ with different acids (1 : 1 in mole ratio) in DCM solution at 1.0 × 10^−3^ mol L^−1^ concentration under 318 nm UV excitation. (e) Images of TRZ and TRZ with different acids (1 : 1 in mole ratio) in DCM solution at 1.0 × 10^−3^ mol L^−1^ concentration under 365 nm UV.

The phenomena triggered our curiosity about how the acid regulates the absorption and emission of TRZ solution. To answer this question, a series of acids with different acidities (p*K*_a_) were added to the 1.0 × 10^−3^ mol L^−1^ DCM solution to explore their roles (Fig. 2c and d). The addition of acetic acid (AcOH) caused no change in absorption (Fig. 2c, black line), while the addition of trifluoroacetic acid (TFA) exhibited a similar phenomenon to ESA (Fig. 2c, red solid line) but with slightly weaker absorption (Fig. 2c, blue solid line) in the region from 390 to 500 nm. In addition, as the acid strength decreased (p*K*_aESA_ = −1.61 (ref. 35) < p*K*_aTFA_ = 0.52 (ref. 36) < p*K*_aAcOH_ = 4.84 (ref. 37)), the intensity of the CT peak decreased accordingly. Interestingly, the emission of TRZ slightly red-shifted from 403 to 414, 507, and 529 nm with the addition of AcOH (Fig. 2d, blue), TFA (Fig. 2d, yellow), and ESA (Fig. 2d, orange), respectively. Comparing the lifetime decays (Fig. S3–S6) at room temperature, the steady state emission at room temperature and at 77 K and the delayed emission at 77 K (Fig. S7–S10), it can be concluded that the emission of TRZ in DCM solution is fluorescence rather than phosphorescence. Fig. 2e clearly shows that the emission and the corresponding quantum yield (*Φ*) increased with increasing acidity. The emission of TRZ at 1.0 × 10^−3^ mol L^−1^, serving as the control, indicated the large Stokes shift attributed to the CT process, which was triggered by adding strong acid (TFA and ESA) into the 1.0 × 10^−3^ mol L^−1^ DCM solution rather than by the increased concentration alone. To clarify the interaction between acid and TRZ, ^1^H NMR titration experiments were performed with TRZ and ESA in CDCl3 (Fig. S11). Although the protonation theory is commonly invoked to explain the acid-modulated fluorescence in previous reports,^25–29^ the observed strong concentration effects suggest the interaction between acid and TRZ in DCM solution does not yield a protonation product but another species with weak intermolecular interaction.

The photophysical properties of TRZ in the presence of acid were then explored in the solid state. TRZ powder was mixed and ground thoroughly with *o*-pyridinesulfonic acid (OPS, p*K*_a_ = 1.73, predicted data from SciFinder), TFA, and ESA with a mole ratio of 1 : 1 to construct powder samples TRZ-OPS, TRZ-TFA, and TRZ-ESA, respectively. The morphology and homogeneity of the ground samples were confirmed as homogeneous amorphous powders under optical microscopy and polarized optical microscopy (Fig. S12). Unlike the single emission of TRZ at 405 nm (Fig. 3a, black), TRZ-OPS exhibited dual emission peaking at 401 and 528 nm (Fig. 3a, green). When mixed with a stronger acid TFA or ESA, the emission of TRZ-TFA (Fig. 3a, yellow) and TRZ-ESA (Fig. 3a, orange) at around 400 nm disappeared, and another emission was observed, peaking at 563 and 585 nm, respectively. Time-resolved PL spectra showed increasing *τ* from 2.40 µs of TRZ-OPS at 528 nm to 5.17 µs of TRZ-TFA at 563 nm and then to 7.53 µs of TRZ-ESA at 585 nm, as shown in Fig. 3b. To further confirm the phosphorescence characteristics of TRZ-OPS (Fig. S13–S14), TRZ-TFA (Fig. S15–S16), and TRZ-ESA (Fig. 3c), the steady-state and delayed emission spectra were measured at 77 K. Here, TRZ-ESA was selected as an example due to the similar results observed for solid samples. In addition to the ambient emission at 585 nm (Fig. 3c, black line), TRZ-ESA exhibited a dual emission at 500 and 577 nm (Fig. 3c, red line) at 77 K. The emission at 500 nm disappeared under a 1 µs delay at 77 K, identifying its fluorescence characteristics and the remaining persistent emission at 577 nm as phosphorescence (Fig. 3c, blue line), which is consistent with our previous report.^34^ To further exclude a thermally activated delayed fluorescence (TADF) mechanism, temperature-dependent lifetime decays of TRZ-ESA (Fig. S17a), TRZ-TFA (Fig. S17b), and TRZ-OPS (Fig. S17c) were measured and the fitting results were summarized (Table S1). The decrease in *τ* with increasing temperature follows the lifetime decay kinetics of phosphorescence, as opposed to that of TADF. Additionally, after being annealed at 150 °C for 10 minutes and followed by cooling to ambient temperature, the phosphorescence emission of TRZ-OPS was red-shifted to 543 nm with the *Φ*_P_ increasing to 6.71% and *τ* extending to 8.29 µs, while the fluorescence emission was almost quenched (Fig. S18 and S19). Hence, the phosphorescence wavelengths of TRZ-OPS, TRZ-TFA, and TRZ-ESA were red-shifted with increasing acidity, demonstrating the tunability of the phosphorescence wavelength using acids of different strengths. The correlation between the acidity and the phosphorescence emission red-shift was further verified by addition of two acids benzenesulfonic acid (BS) and *p*-toluenesulfonic acid which have a similar structure (Fig. S20).

**Fig. 3:**
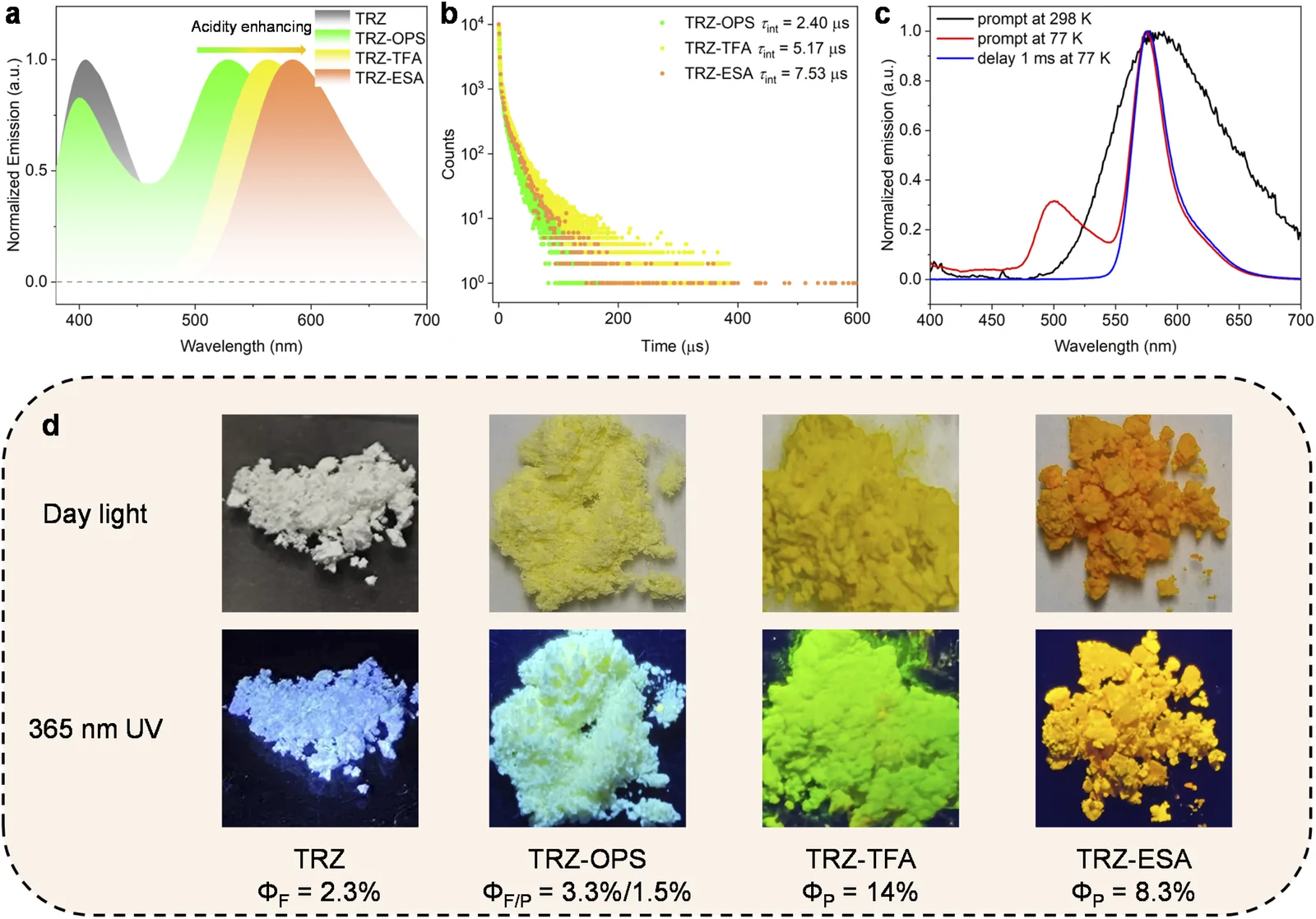
Photophysical properties of powder samples. (a) Normalized emission of TRZ, TRZ-OPS, TRZ-TFA, and TRZ-ESA under 318 nm UV excitation. (b) Time-resolved PL spectra of TRZ-OPS, TRZ-TFA, and TRZ-ESA at 528 nm, 563 nm, and 585 nm, respectively. (c) Normalized emission of TRZ-ESA at 298 K and 77 K, and delayed emission for 1 ms at 77 K. (d) Images of TRZ, TRZ-OPS, TRZ-TFA, and TRZ-ESA under daylight and 365 nm UV irradiation.

The images of TRZ, TRZ-OPS, TRZ-TFA, and TRZ-ESA under daylight and 365 nm UV, along with their *Φ*_F_ and *Φ*_P_, are shown in Fig. 3d. The phosphorescence properties of powder samples are also summarized in Table 1 and the lifetime fitting details are provided in Table S2.

**Table 1:** Summary of photophysical properties of powder samples^a^

Sample	*λ*_P_ (nm)	*τ*_P_ (µs)	*Φ*_P_ (%)
TRZ-OPS	528/543^b^	2.40/8.29^b^	1.52/6.71^b^
TRZ-TFA	563	5.17	14.05
TRZ-ESA	585	7.53/85.2^c^	8.33

a All samples were measured with 318 nm UV excitation.

b After being annealed at 150 °C for 10 minutes and cooled to room temperature.

c With 100 µs of gating.

Doping organic phosphorescent molecules in a polymer matrix is an efficient method to stabilize the triplet excited state and thus to enhance the phosphorescence.^32,38^ Here, after exploring the photophysical properties of powders, TRZ and TRZ-ESA were doped in polymethyl methacrylate (PMMA) to prepare PMMA films of TRZ-P and TRZ-ESA-P, respectively, which were investigated to further explore the role of ESA in triggering RTP. Although the TRZ-P film is transparent, the TRZ-ESA-P film is opaque, indicating that TRZ molecules are partly aggregated in the matrix rather than mono-dispersed in the presence of ESA (Fig. 4a). TRZ-ESA-P showed photoactivated RTP within 4 s under 370 nm irradiation, while the phosphorescence strength slightly decreased with the UV irradiation continuing to 12 s (Fig. 4b). Although TRZ-P exhibited weak RTP at 550 nm with a *τ* of 64.9 µs and blue fluorescence at 400 nm with a *τ* of 1.96 ns (Fig. 4c, black line and Fig. S21 and S22), which indicated the contribution of the polymer matrix, the strong RTP of TRZ-ESA-P at 538 nm with a *τ* of 304 µs and *Φ* of 5.93% demonstrated the major role of ESA in stabilizing RTP (Fig. 4c, red line and Fig. S23).

**Fig. 4:**
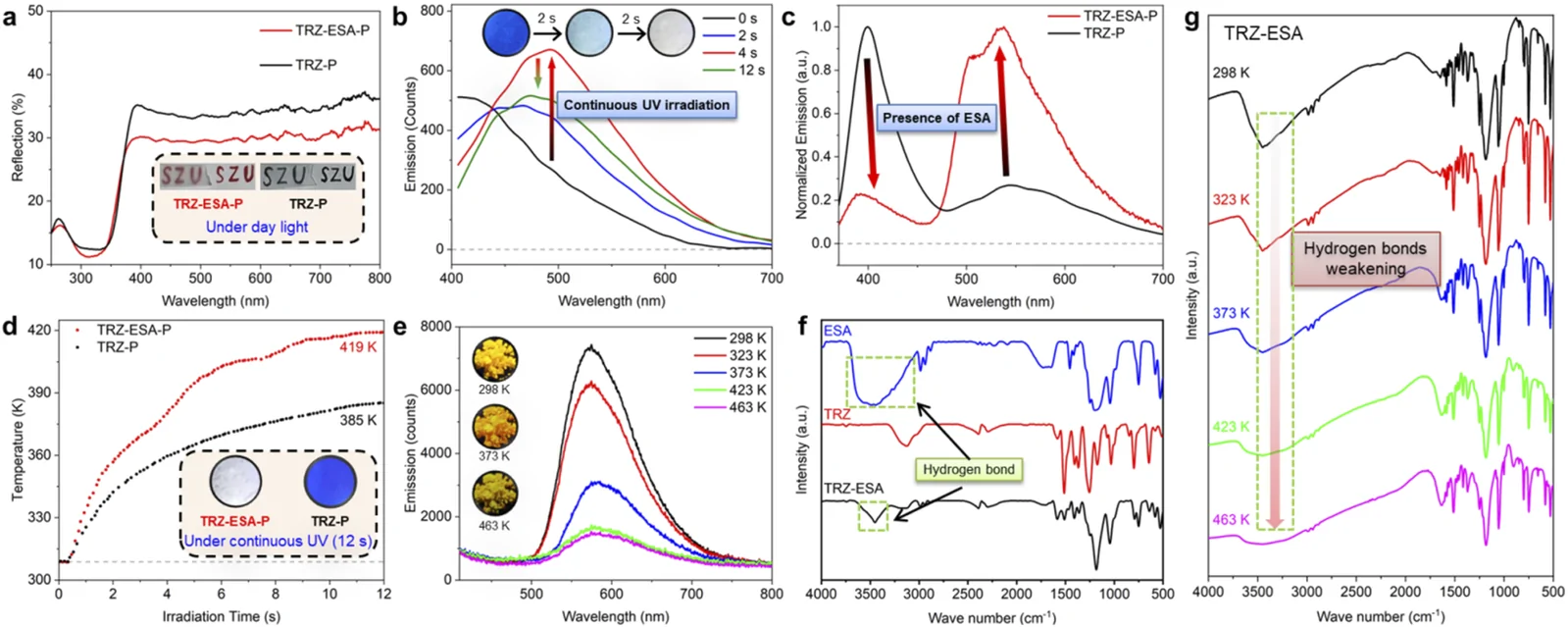
HB triggered photothermal and phosphorescent properties in doped PMMA films and powder samples. (a) Diffuse reflection spectra and images under daylight of TRZ-ESA-P and TRZ-P. (b) On-line emission of TRZ-ESA-P under continuous 370 nm UV irradiation for different durations. Insets show images of TRZ-ESA-P under 370 nm UV with the irradiation duration indicated. (c) Steady state emission of TRZ-ESA-P and TRZ-P under 318 nm excitation. (d) Photothermal effect of TRZ-ESA-P and TRZ-P with 370 nm UV irradiation (290 mW cm^−2^). Insets show images of TRZ-ESA-P and TRZ-P under continuous 370 nm UV irradiation for 12 s. (e) Emission of TRZ-ESA under 370 nm excitation at different temperatures. Insets show images of TRZ-ESA under 370 nm UV with the temperature indicated. (f) FTIR spectra of ESA, TRZ, and TRZ-ESA at room temperature. (g) FTIR spectra of TRZ-ESA at different temperatures.

Besides, the phosphorescence of TRZ-ESA-P decreases under continuous UV irradiation which attracted our attention (Fig. 4b, red and green lines). The photothermal effect of TRZ was reported in our previous work, which drove us to explore the photothermal effect of doped PMMA films. The temperature of TRZ-ESA-P was raised to 419 K in 12 s (Fig. 4d, red dots) with the phosphorescence emission maintained (Fig. 4d inset, left). Meanwhile, TRZ-P exhibited a significantly weaker photothermal effect than TRZ-ESA-P, with temperature raised to 385 K in 12 s (Fig. 4d, black dots) and no photoactivated phosphorescence observed (Fig. 4d inset, right) under the same UV irradiation conditions. These results indicated that the presence of ESA might be capable of stabilizing the phosphorescence at high temperature. Thus, PL spectra of TRZ-ESA were measured at increasing temperature to verify this hypothesis (Fig. 4e). As the temperature increased from 298 K to 463 K, the phosphorescence intensity of TRZ-ESA gradually decreased while its shape and wavelength remained unchanged. Both TRZ-OPS (Fig. S24) and TRZ-TFA (Fig. S25) exhibited similar temperature-dependent emission except for less stable HTP to TRZ-ESA, with the phosphorescence completely quenched at 463 K and 373 K, respectively. The images of TRZ-ESA under 365 nm UV irradiation were shown in insets with the temperature indicated in Fig. 4e. The emission spectra of TRZ-ESA, TRZ-TFA, and TRZ-OPS before and after heating were compared (Fig. S26). TRZ-ESA exhibited phosphorescence retention after heating, whereas the phosphorescence of TRZ-TFA was almost irrecoverable, and TRZ-OPS showed enhanced phosphorescence. The lower phosphorescence quenching temperature and poor retention of phosphorescence performance after heating of TRZ-TFA were attributed to the volatilization of TFA upon heating (Fig. S27). The phosphorescence enhancement of TRZ-OPS likely originates from a reconstruction of the hydrogen-bonding network during thermal annealing: some of the original intramolecular hydrogen bonds of OPS are disrupted at elevated temperatures, and upon cooling, intermolecular hydrogen bonds between TRZ and OPS are formed, thereby strengthening the phosphorescence emission of TRZ.

The pronounced quenching of phosphorescence at elevated temperatures is primarily attributed to the accelerated non-radiative transitions at high temperature, which explains the scarcity of reported HTP systems.^6–13^ It has been studied in the past few decades that hydrogen bonds in the solid state can retain their stability at high temperatures.^32,39^ Based on the primary inferences from DCM solutions, Fourier transform infrared (FTIR) spectroscopy was applied to investigate the hydrogen bonds between TRZ and acids in the solid state.^40^ Among all the aforementioned acids, ESA exhibits the strongest acidity. Therefore, TRZ-ESA is chosen as the example due to its most easily observable hydrogen bond. The FTIR spectra of ESA, TRZ, and TRZ-ESA were measured at room temperature, as shown in Fig. 4f. The broad and strong peak at around 3500 cm^−1^ of ESA was attributed to the abundant hydrogen bonds between ESA molecules (Fig. 4f, blue line), while in TRZ powder, no hydrogen bond was observed (Fig. 4f, red line). For the sample of TRZ-ESA, a broad peak at 3450 cm^−1^ was observed, which is different from that of ESA, indicating the hydrogen bonds formed between ESA and TRZ molecules (Fig. 4f, black line). The temperature independent FTIR spectra of TRZ-ESA also showed hydrogen bonds even when the temperature was increased to 463 K (Fig. 4g), correlating with the retaining of phosphorescence at increased temperature. In conclusion, HTP can be achieved by the HB strategy.

The SOC values of TRZ (Fig. 5a, left) and TRZ-ESA (Fig. 5a, right) in the DCM environment were calculated to explain the role of the acid triggered phosphorescence. For pure TRZ, the sum of the SOC values of effective intersystem crossing (ISC) channels within the range |*E*_S1_ − *E*_Tn_| < 0.30 eV is 0.830 cm^−1^ (Fig. 5a, left, red), but for the sample of TRZ-ESA, the sum of the effective SOC is increased to 3.239 cm^−1^ by fourfold (Fig. 5a, right, red), indicating that SOC can be boosted by the HB. However, strong non-radiative transitions still hinder the observation of phosphorescence in DCM solution.

**Fig. 5:**
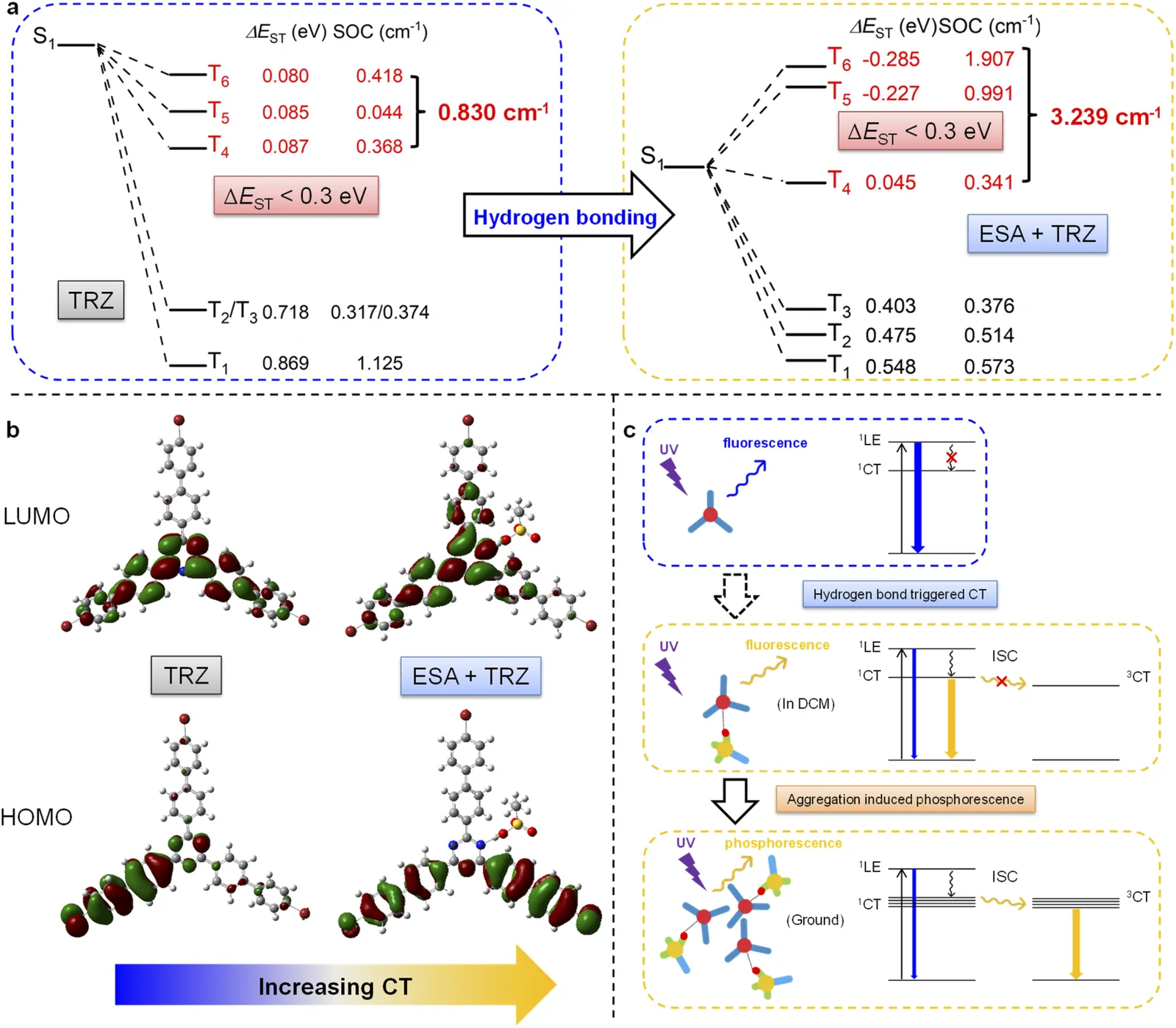
Proposed theoretical mechanism for acid regulated phosphorescence. (a) The SOC boosting through the formation of HB. (b) Frontier molecular orbital profiles of TRZ and TRZ-ESA in DCM solution. (c) The proposed orbital change of TRZ in DCM (top), TRZ-ESA in DCM (middle), and TRZ-ESA in ground powder (bottom).

To better understand the role of the acid in triggering tunable HTP, the frontier molecular orbital profiles of TRZ and TRZ-ESA in DCM solution were investigated using DFT (Fig. 5b). The HOMO is primarily localized on the biphenyl side group for both TRZ and TRZ-ESA, with only a minor contribution from the triazine core, and this contribution is further reduced in TRZ-ESA (Fig. 5b, bottom). The ESA group not only reduces the HOMO contribution of the triazine core, but also increases the LUMO contribution on the same moiety. This change in orbital distribution results in an enhanced CT character, which is positively correlated with the acidity, as proven using the absorption spectra under different acid additions (Fig. 2a and c).

Based on the above conclusion, a simplified Jablonski diagram was proposed, as shown in Fig. 5c. In pure TRZ dilute solution, the excited state is mainly a ^1^LE state due to the relatively close electronegativity of the triazine core and the biphenyl side group, which results in the intrinsic blue emission (Fig. 5c, top). In concentrated solution, HB between the triazine core and the acid increases the electronegativity of triazine, leading to the turn-on of a ^1^CT state (Fig. 5c, middle). Consequently, the emission switches from the ^1^LE band to the ^1^CT band. Due to the electrostatic-interaction-enhanced phosphorescence, hydrogen-bonding-suppressed nonradiative decay, and π-halogen-bonding-enhanced ISC, aggregation is an effective strategy to induce phosphorescence in organic small molecules.^41–44^ In ground samples such as TRZ-ESA, TRZ molecules in the amorphous state exhibit different conformations. In such aggregates, the close intermolecular contact and orbital coupling among TRZ molecules provides additional intermolecular and intramolecular ISC pathways from the ^1^CT to ^3^CT state across varying conformations, thereby effectively enhancing the overall SOC of the material and ultimately triggering phosphorescence (Fig. 5c, bottom). The energy level of the ^3^CT state as a polarized state can be further decreased by the increased strength of the hydrogen bond, resulting in tunable phosphorescence wavelengths that are related to the acidity.

## Conclusions

In summary, a series of wavelength-tunable and high-temperature-tolerant phosphorescent materials were developed based on a triazine derivative (TRZ) and different strengths of acids. Contrary to the classical protonation mechanism conventionally assumed for acid-controlled phosphorescence, and in correction of the mechanistic assignment in our prior reports, we demonstrate that hydrogen bonding (HB) between the triazine core and the acid is the key interaction governing the observed photophysical behavior. The HB mechanism enables not only fine-tuning of the phosphorescence wavelength through modification of the anionic moiety but also the stabilization of phosphorescence at elevated temperatures up to 463 K. More importantly, by demonstrating that stable HB complexes can form even with strong acids (p*K*_a_ < 0) instead of protonated products, this work extends the HB-based regulation strategy from conventionally explored weak acid or neutral systems into the strong acid territory. Compared with existing HTP systems mostly relying on the confinement strategy based on host–guest doping, this HB strategy required only simple mixing and grinding, thereby greatly simplifying the preparation process (Table S3). Meanwhile, after doping in a PMMA matrix, the maintained phosphorescence with the temperature rising to 419 K by the photothermal effect proved the HTP stability of this HB strategy. Given the modular nature of HB interactions and the broad availability of HB donors and acceptors, we anticipate that this strategy can be readily extended to other luminophore systems through rational molecular design, providing a versatile platform for developing tunable organic HTP materials. This work provides insight into developing tunable organic HTP materials and explores their potential prospects in optoelectronic devices operating at high temperature.

## Author contributions

C.-Z. Z., B. Z. T. and Z.-Q. Y. conceived the project. S.-Q. Q., J.-R. C., M.-Z. L. and A. H. performed the experiments, S.-Q. Q., J.-R. C., C.-Z. Z., B. Z. T. and Z.-Q. Y. analyzed the experimental data. J.-R. C. and Y. X. performed the theoretical calculations. S.-Q. Q. wrote the manuscript. S.-Q. Q., J.-R. C., C.-Z. Z., B. Z. T. and Z.-Q. Y. discussed the results and contributed to the preparation of the final manuscript.

## Conflicts of interest

There are no conflicts to declare.

## Supplementary Material

SC-017-D6SC03982K-s001

## Data Availability

All data supporting this study, including detailed methods, experimental details, DFT calculations, and photophysical property studies, are available in the manuscript and supplementary information (SI). All data are available upon request. Supplementary information is available. See DOI: https://doi.org/10.1039/d6sc03982k.
