# Supplementary material for: Hydrogen bonding triggered tunable high temperature phosphorescence
Source: Chem Sci. 2026 Aug 5;17(38):18454–61. doi: 10.1039/d6sc03982k (PMC13473997; doi:10.1039/d6sc03982k)
Supplement: Supplementary file 1 [file SC-017-D6SC03982K-s001.pdf]

Supplementary Information (SI) for Chemical Science.
This journal is © The Royal Society of Chemistry 2026

Supporting information

Hydrogen Bonding Triggered Tunable High Temperature Phosphorescence

Sheng-Qi Qiu‡,[a] Jun-Ran Chen‡,[a] Yao Xiao,[a] Ming-Zhu Liao,[a] Anqi He,[b] Cai-Zhen Zhu*,[a] Ben Zhong Tang*,[c] Zhen-Qiang Yu*[a]

‡These authors contributed equally.

[a] Dr. S.-Q. Qiu, J.-R. Chen, Y. Xiao, M.-Z. Liao, Prof. Dr. C.-Z. Zhu, Prof. Dr. Z.-Q. Yu
College of Chemistry and Environmental Engineering
Shenzhen University
Shenzhen 518071, China
E-mail: zqyu@szu.edu.cn, czzhu@szu.edu.cn

[b] Dr. A. He
Beijing National Laboratory for Molecular Sciences, College of Chemistry and Molecular Engineering
Peking University
Beijing 100871, P. R. China

[c] Prof. Dr. B. Z. Tang
Guangdong Basic Research Center of Excellence for Aggregate Science, School of Science and Engineering
The Chinese University of Hong Kong
Shenzhen 518172, China
E-mail: tangbenz@cuhk.edu.cn

List of Contents

1. **General information** 3
2. **Synthetic route** 4
3. **Supporting figures and tables** 5
4. **DFT calculations** 20
5 **References** 27

1. General information

Chemicals were purchased from commercial suppliers (J&K and Sigma Aldrich) and used without further purification unless otherwise stated. Analytical thin layer chromatography (TLC) was performed on precoated silica gel HSGF 254 plates. Flash column chromatography was performed using Tsingtao silica gel (60, particle size 0.040-0.063 mm). Visualization on TLC was achieved by use of UV light (254 nm and 365 nm). ^{1}H and ^{13}C spectra were recorded on Bruker 500

or 600 MHz spectrometer in $CDCl_3$, DMSO-d_6 or methanol-d_4, with tetramethylsilane (TMS) as internal standard. ^{31}P NMR spectra were recorded on Bruker 500 MHz spectrometer in DMSO-d_6, with 85% phosphoric acid solution in water as external standard. The chemical shifts are expressed in ppm and coupling constants are given in Hz. Data for ^{1}H NMR is recorded as follows: chemical shift (δ, ppm), multiplicity (s = singlet; d = doublet; t = triplet; q = quartet; m = multiplet; br = broad), coupling constant (Hz), integration. Data for ^{13}C NMR is reported in terms of chemical shift (δ, ppm), multiplicity (s = singlet; d = doublet), coupling constant (Hz). High resolution mass spectrometry (HRMS) analyses were performed at a Thermo Q-Exactive Focus Inc mass instrument (APCI). Ultraviolet absorption spectrum was measured using Shimadzu UV-2550. Fluorescence emission spectrum, lifetime, and quantum efficiency were measured using Edinburgh FS5. Infrared Spectroscopy (IR) was measured using SHIMADZU IR Affinity-1.

2. Synthetic route

Synthesis of p-Br-TRZ

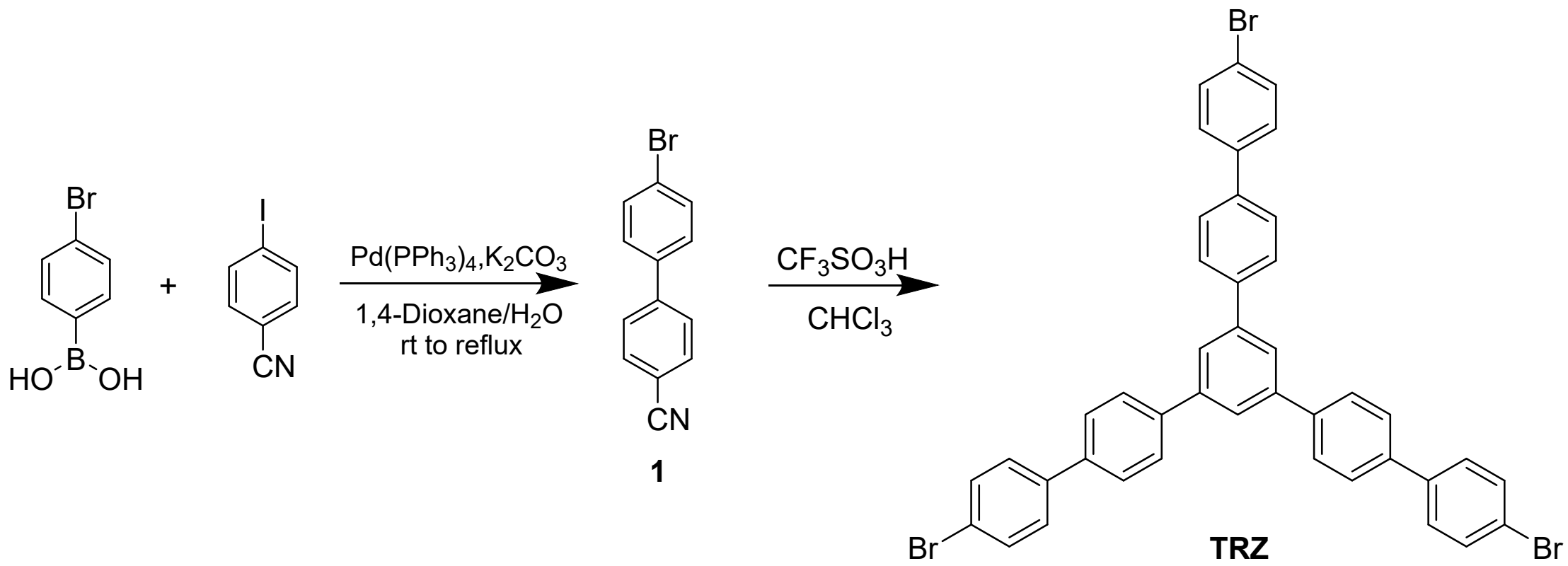

Synthesis of 1[33]

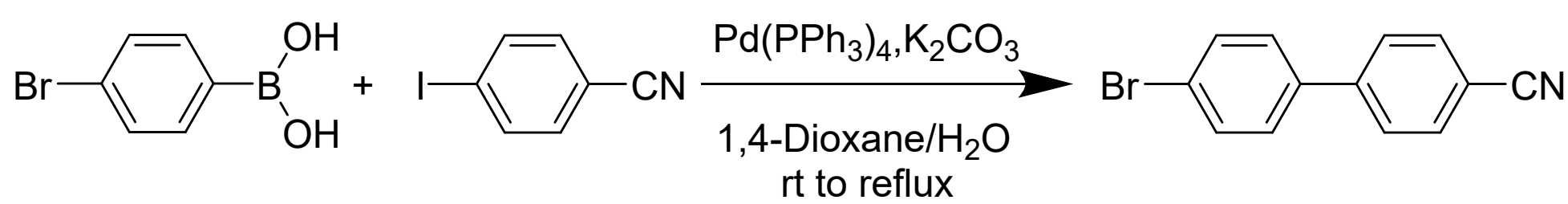

Compound **1** was synthesized following the procedure reported in the article[33].

Synthesis of TRZ[34]

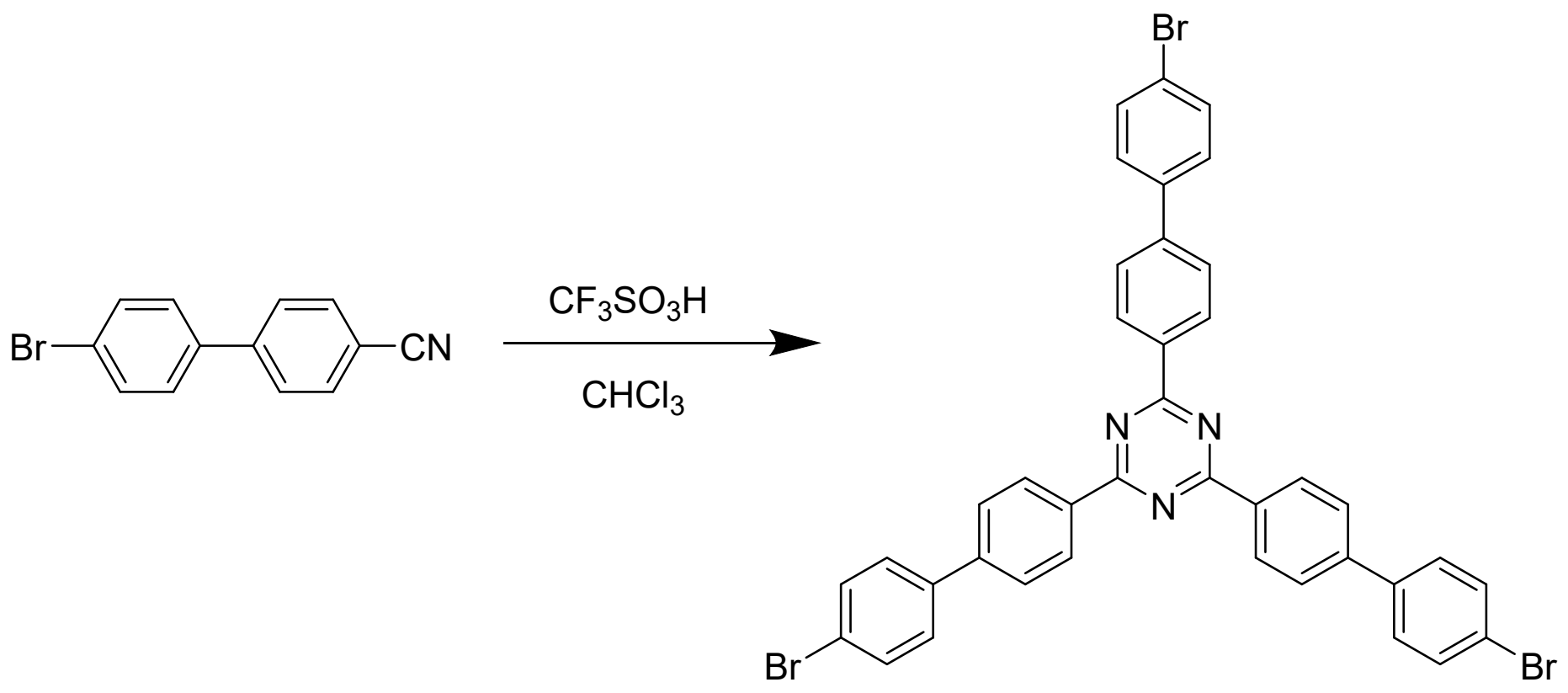

Compound **TRZ** was synthesized following the procedure reported in the article[33].

3. Supporting figures and tables

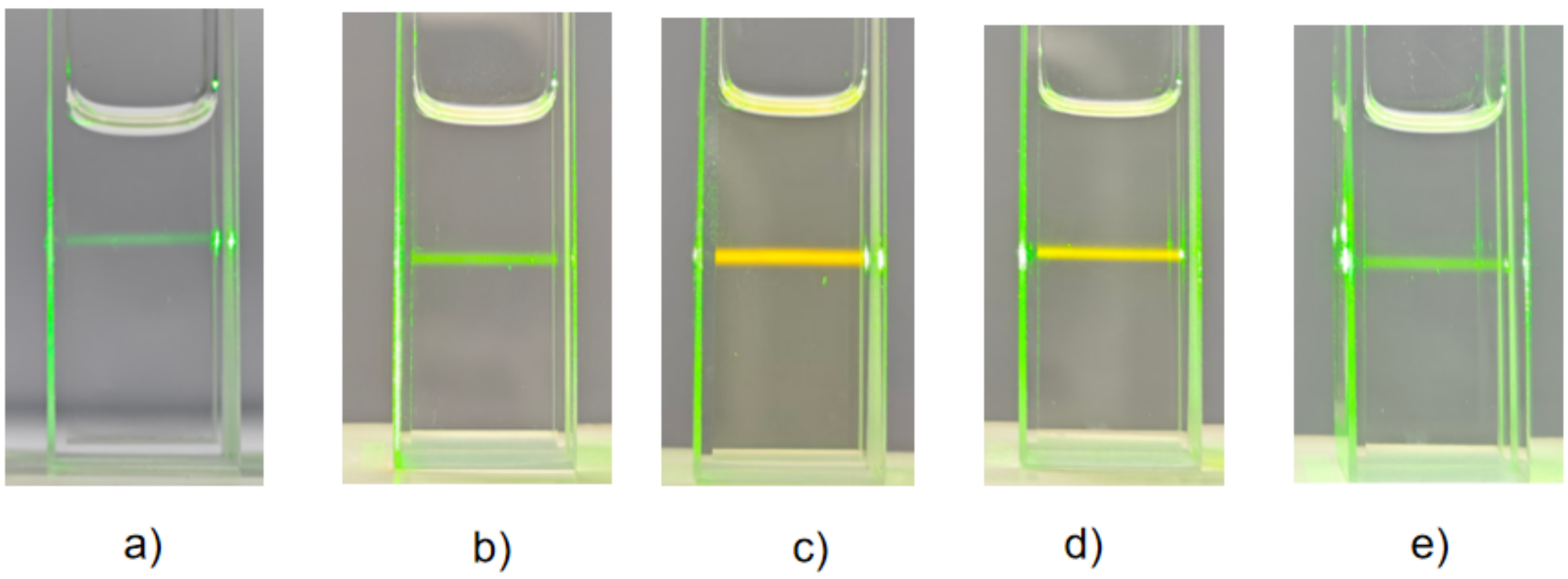

Figure S1. Unless otherwise stated, all solution samples were filtered by PTFE filter membrane with pore size of 0.22 μm. Testing for the Tyndall effect of a) DCM, solution of b) TRZ, c) TRZ with ESA, d) TRZ with TFA, and e) TRZ with AcOH.

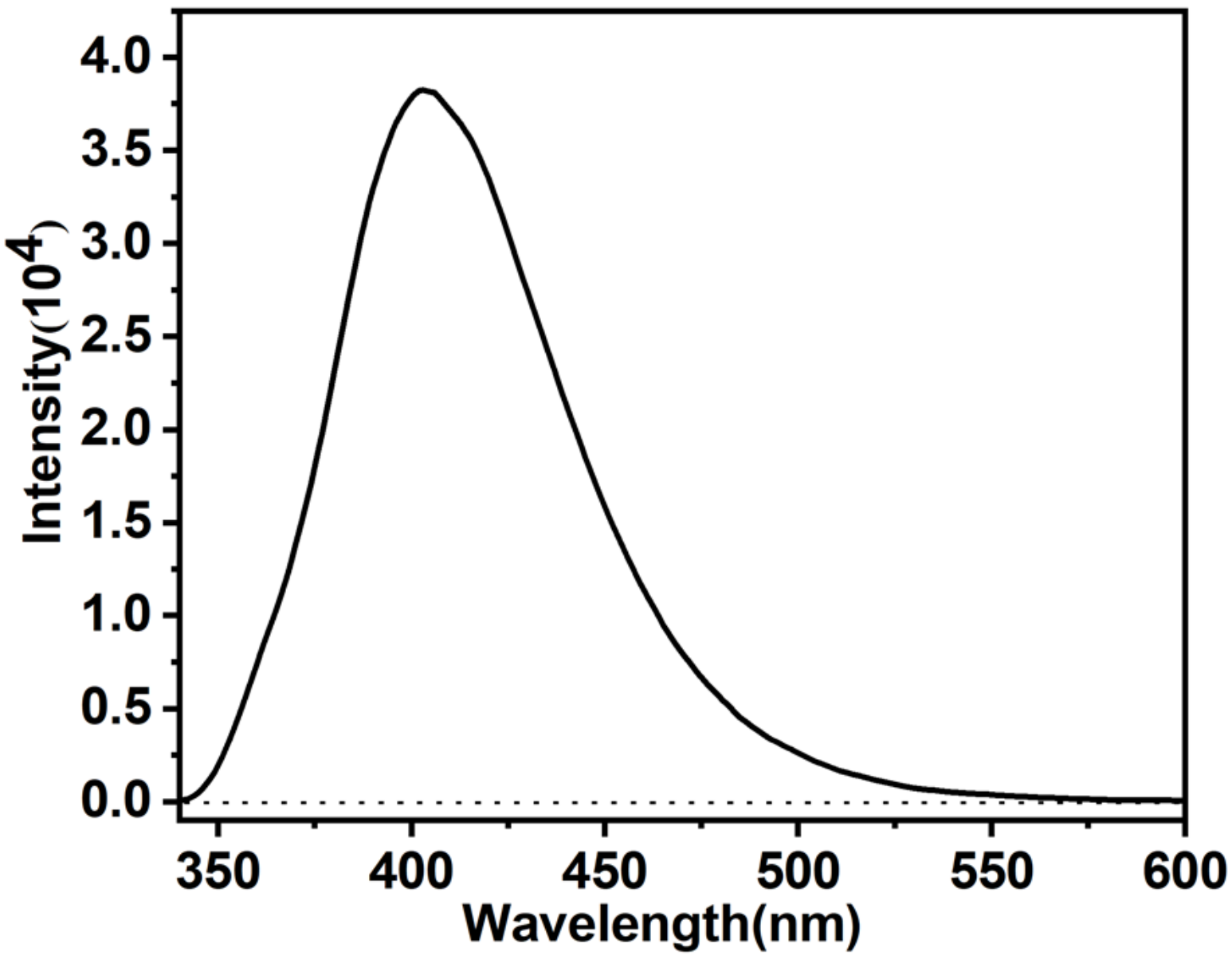

Figure S2. Emission spectra of **TRZ** in DCM. (λ_{ex} = 318 nm，c = 1.0×10^{-3} mol L^{-1})

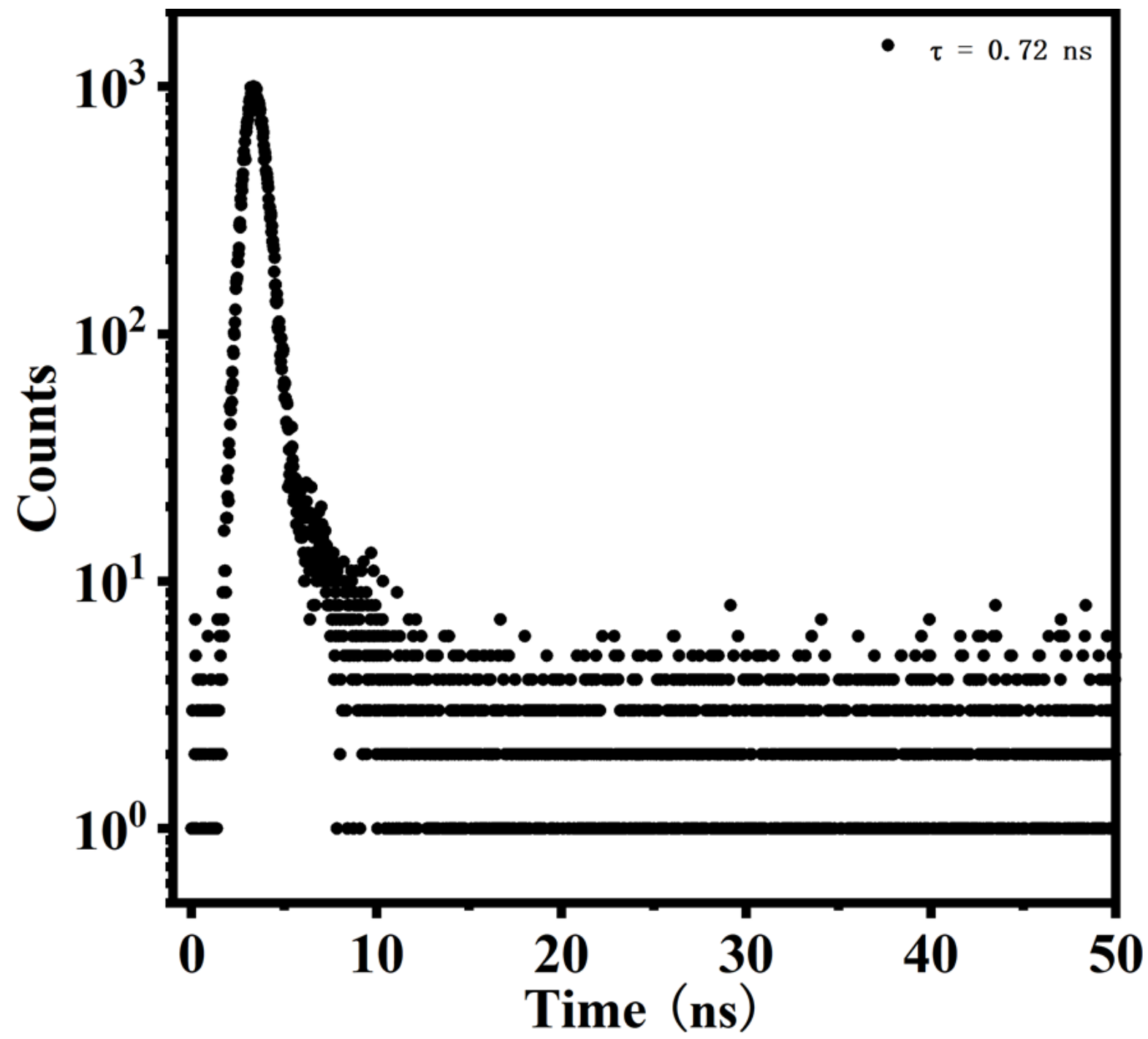

Figure S3. Time-resolved decay curves of **TRZ** in DCM.

(λ_{ex} = 318 nm，λ_{em} = 405 nm，c = 1.0×10^{-3} mol L^{-1})

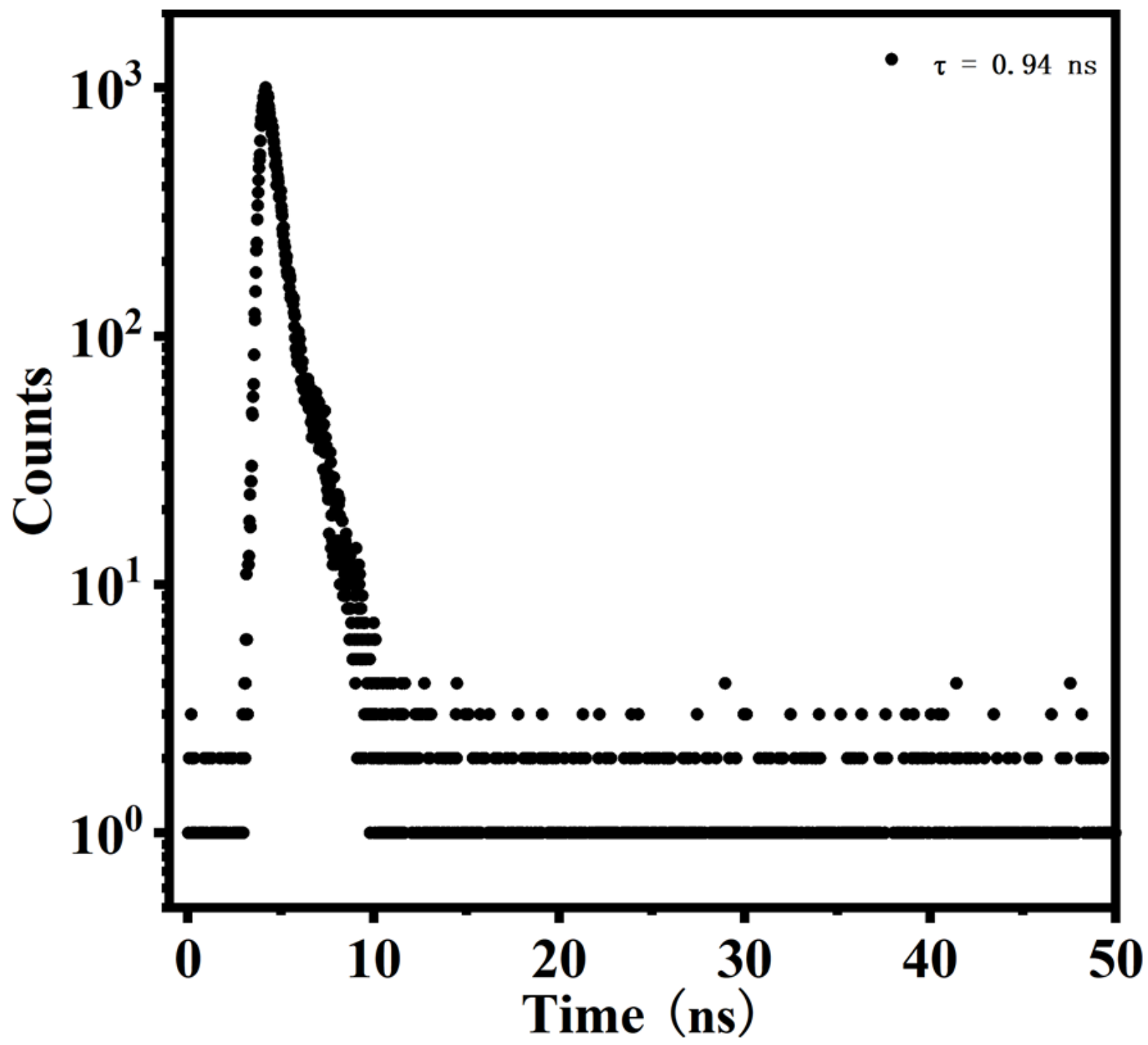

Figure S4. Time-resolved decay curves of **TRZ with AcOH** in DCM.

(λ_{ex} = 318 nm, λ_{em} = 415 nm, c = 1.0×10^{-3} mol L^{-1})

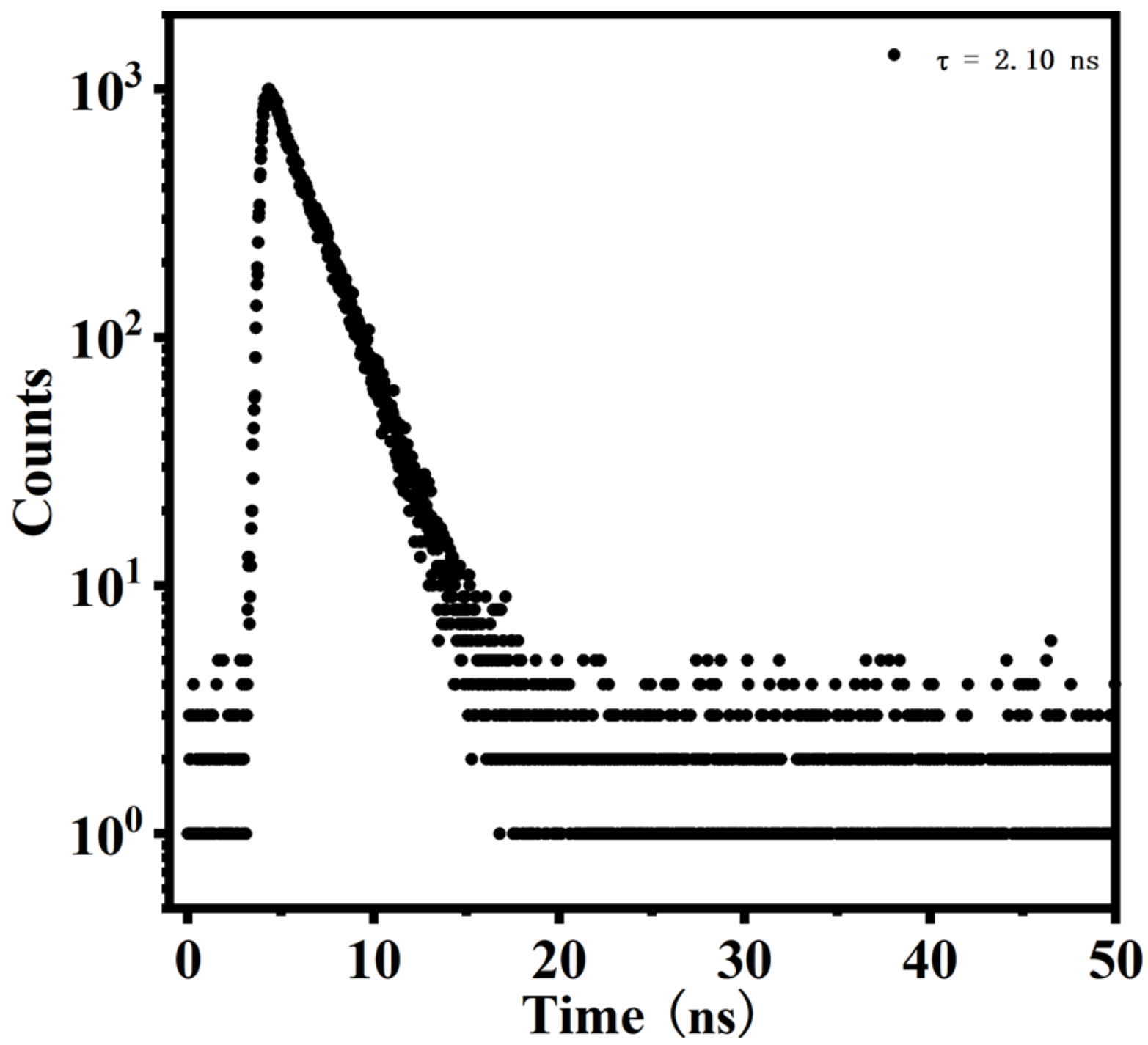

Figure S5. Time-resolved decay curves of **TRZ with TFA** in DCM.

(λ_{ex} = 318 nm，λ_{em} = 515 nm，c = 1.0×10^{-3} mol L^{-1})

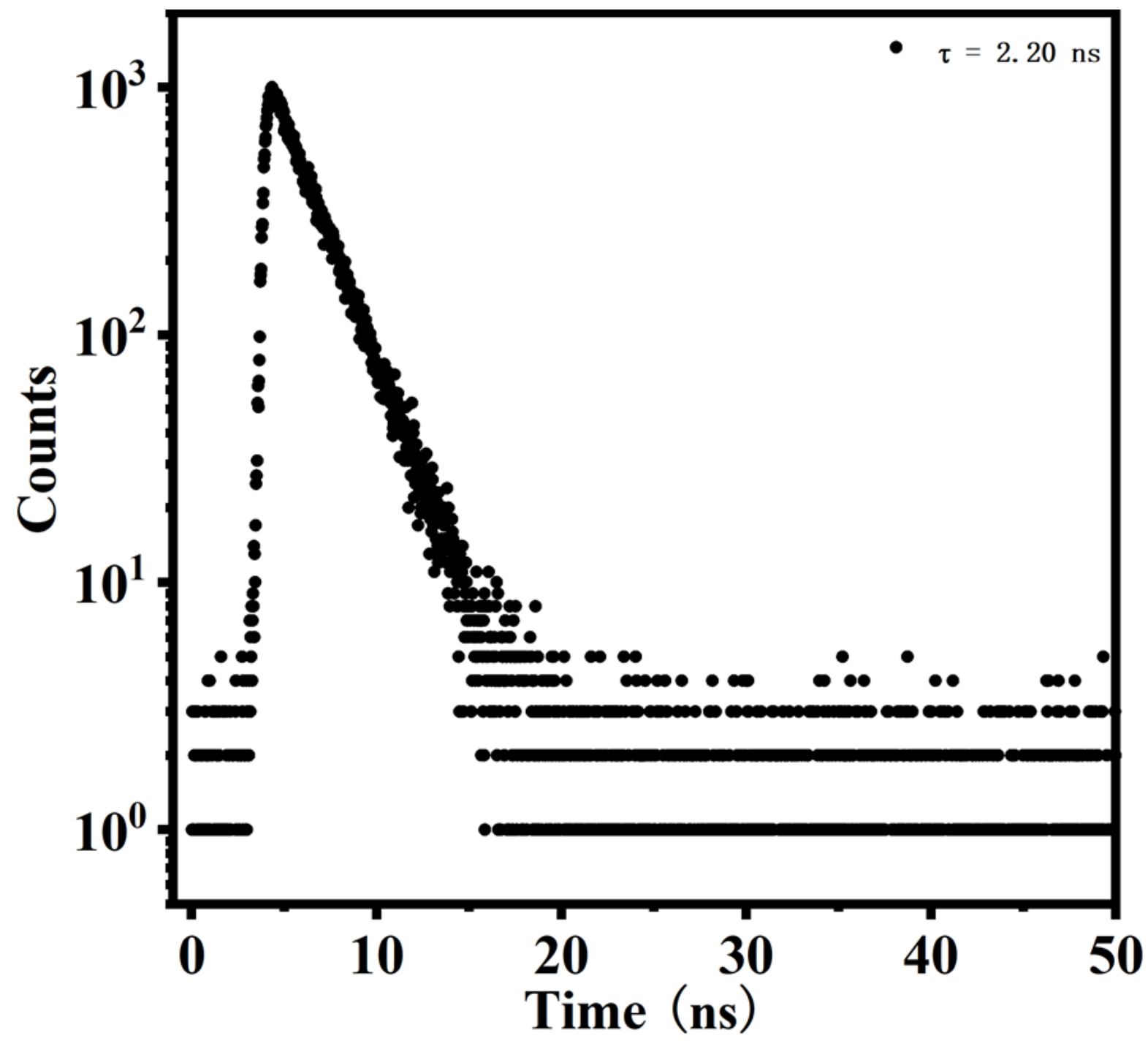

Figure S6. Time-resolved decay curves of **TRZ with ESA** in DCM.

(λ_{ex} = 318 nm，λ_{em} = 550 nm，c = 1.0×10^{-3} mol L^{-1})

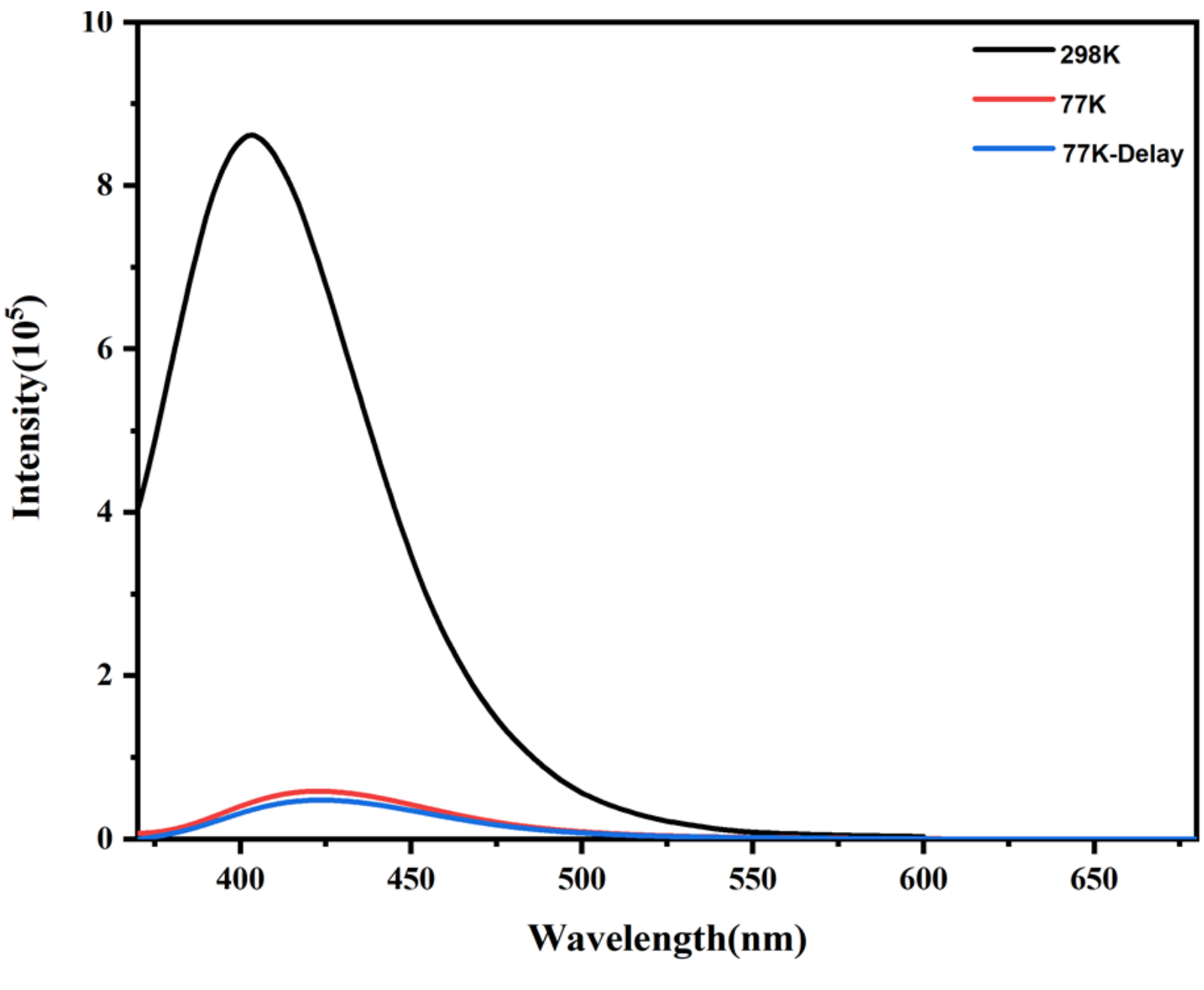

Figure S7. Steady-state PL spectra at 298 K and 77 K, phosphorescence spectra (delay 1 ms) at 77 K of **TRZ** in DCM.(λ_{ex} = 318 nm，c = 1.0×10^{-3} mol L^{-1})

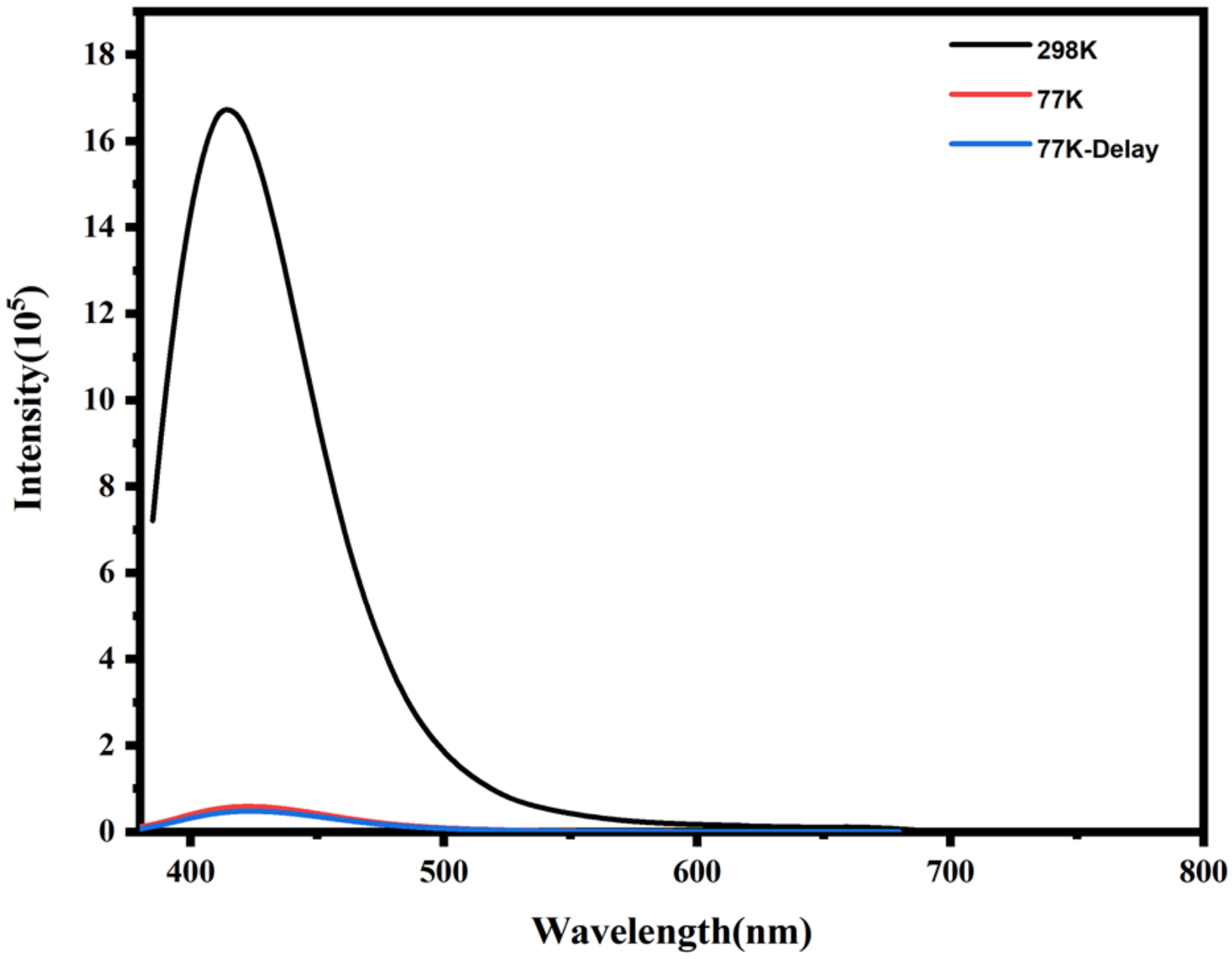

Figure S8. Steady-state PL spectra at 298 K and 77 K, phosphorescence spectra (delay 1 ms) at 77 K of **TRZ with AcOH** in DCM. (λ_{ex} = 318 nm，c = 1.0×10^{-3} mol L^{-1})

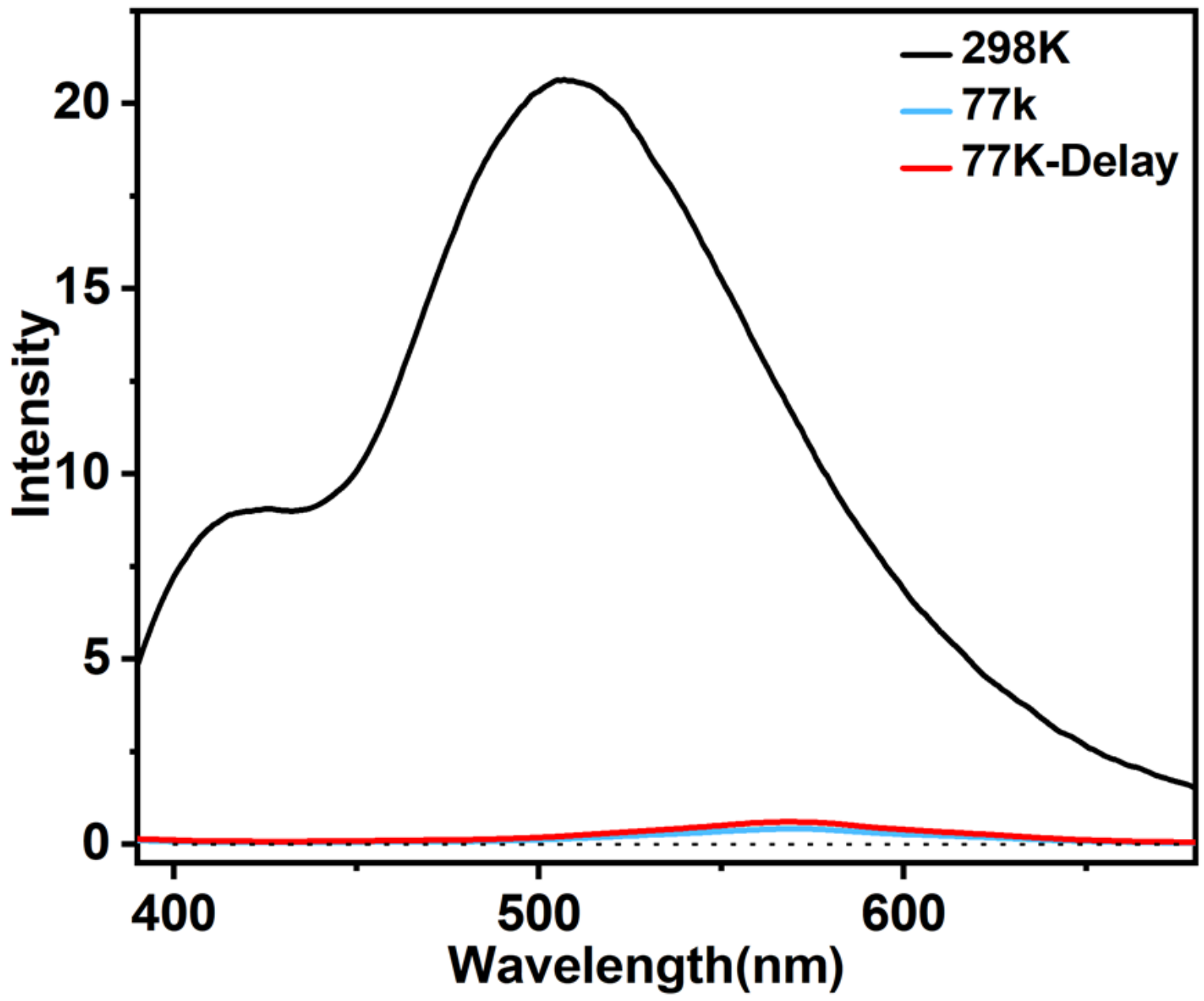

Figure S9. Steady-state PL spectra at 298 K and 77 K, phosphorescence spectra (delay 1 ms) at 77 K of **TRZ with TFA** in DCM. (λ_{ex} = 318 nm，c = 1.0×10^{-3} mol L^{-1})

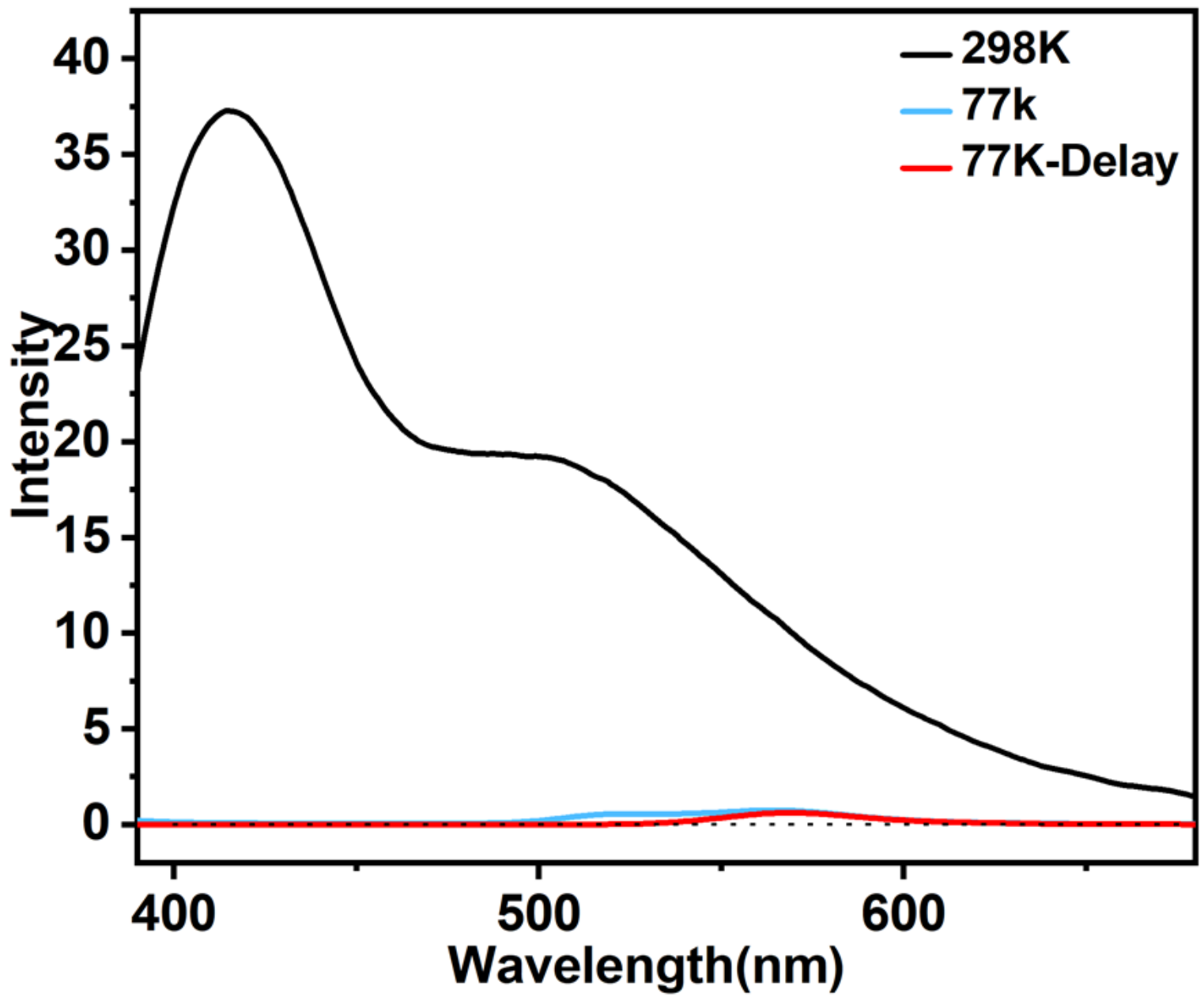

Figure S10. Steady-state PL spectra at 298 K and 77 K, phosphorescence spectra (delay 1 ms) at 77 K of **TRZ with ESA** in DCM. (λ_{ex} = 318 nm，c = 1.0×10^{-3} mol L^{-1})

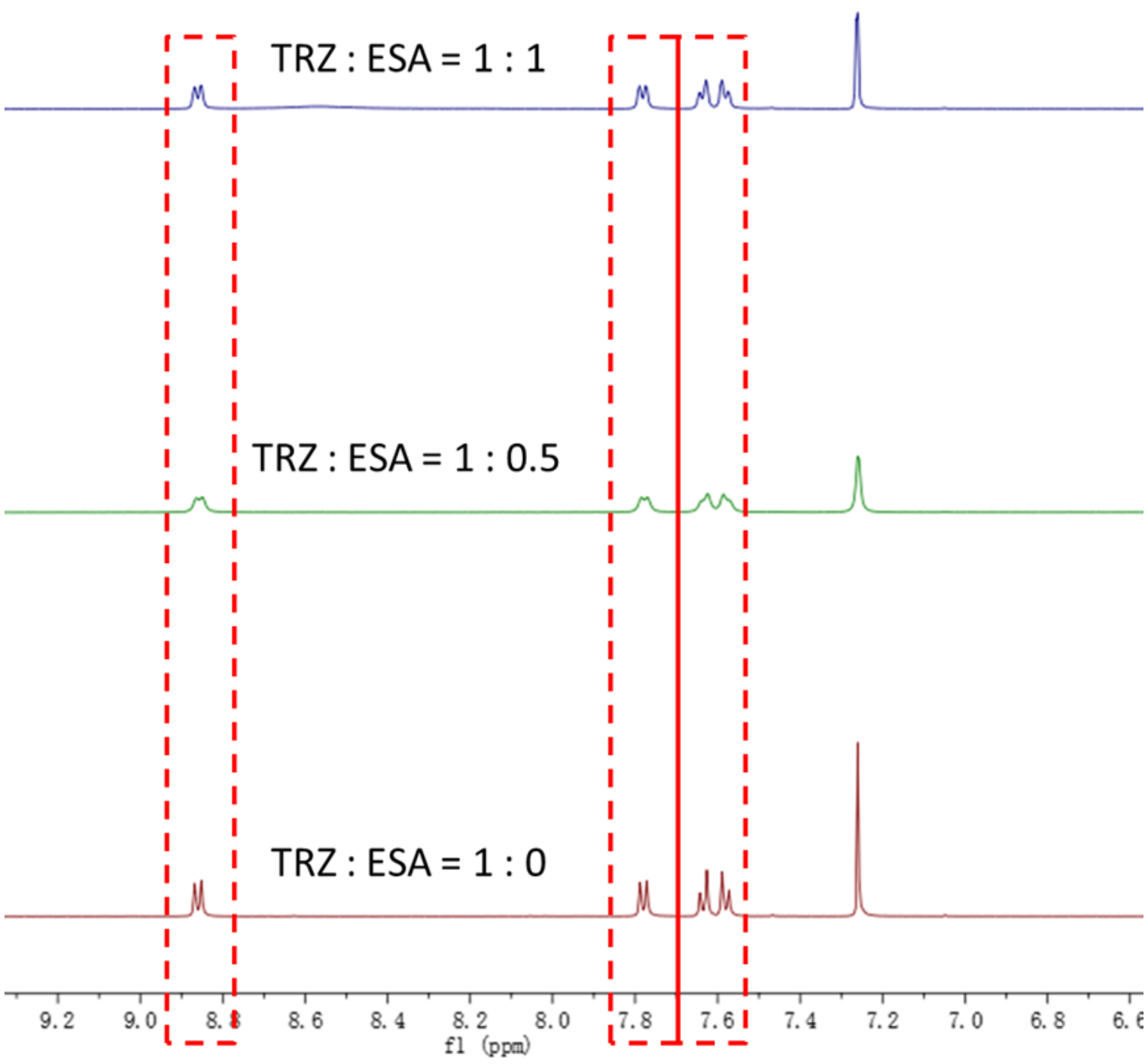

Figure S11. ^{1}H NMR titration experiments.

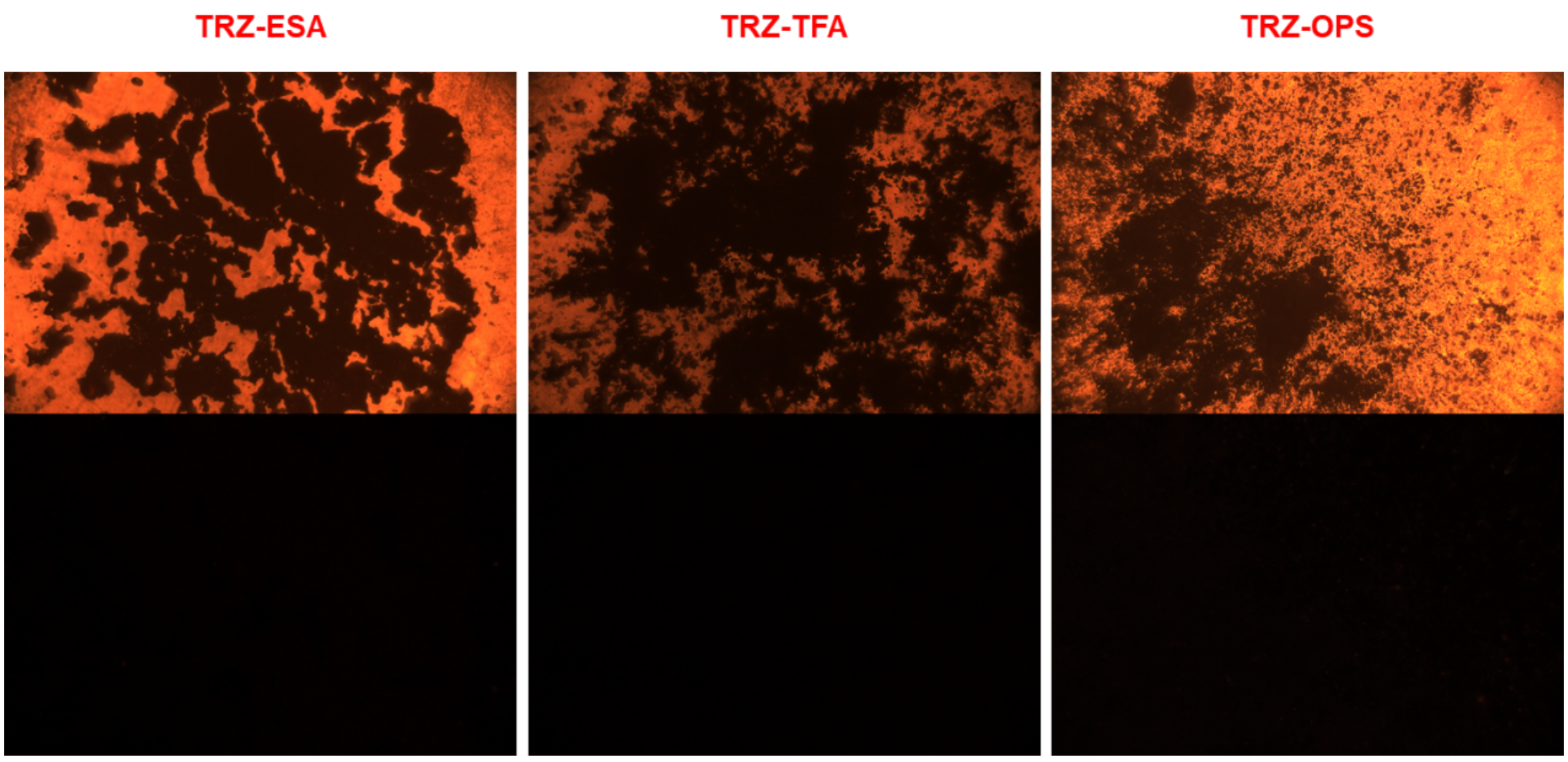

Figure S12. Optical microscopy (up) and the polarized optical microscopy (bottom) images of same positions.

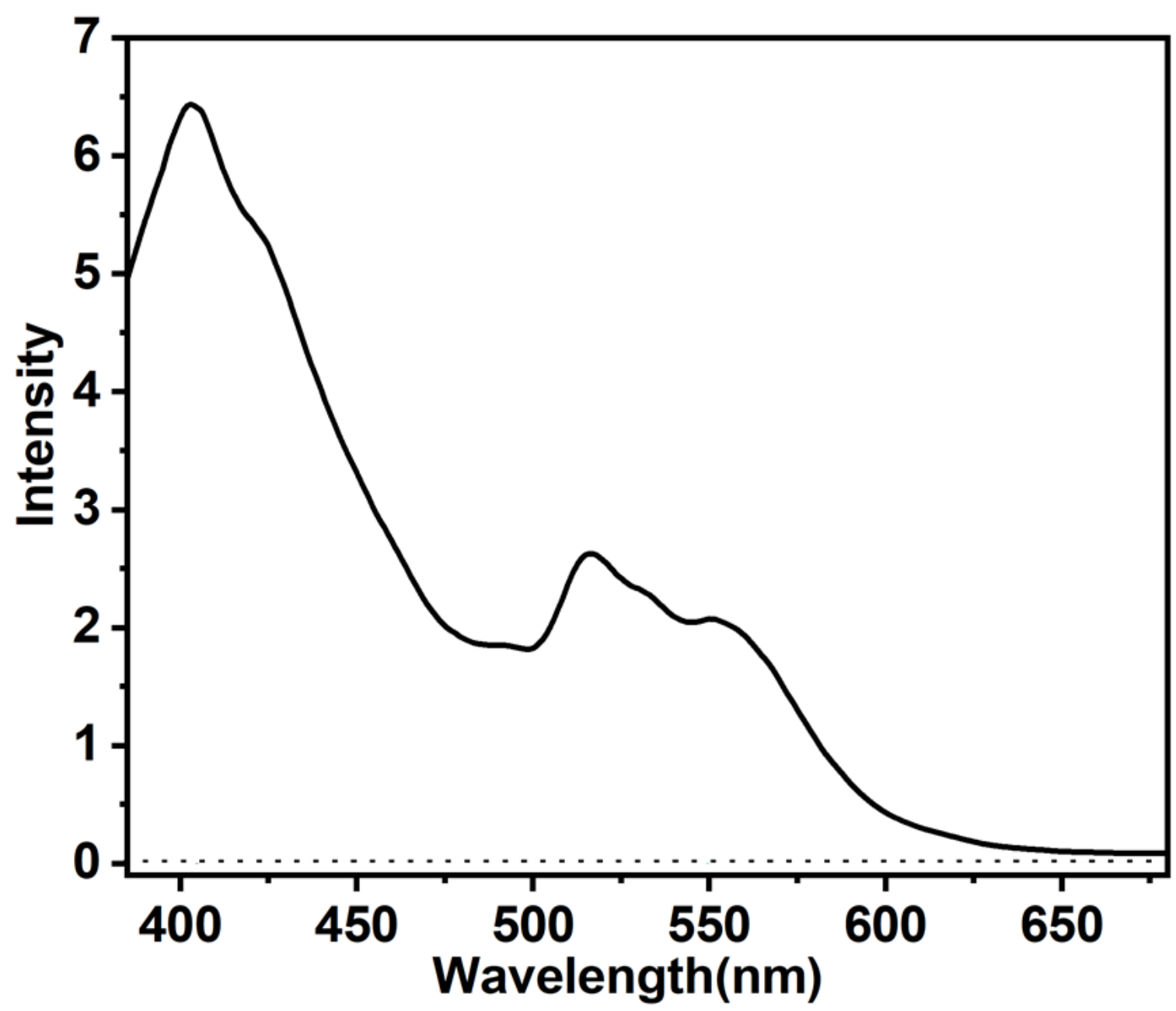

Figure S13. Steady-state PL spectra of **TRZ-OPS** at 77K (λ_{ex} = 318 nm).

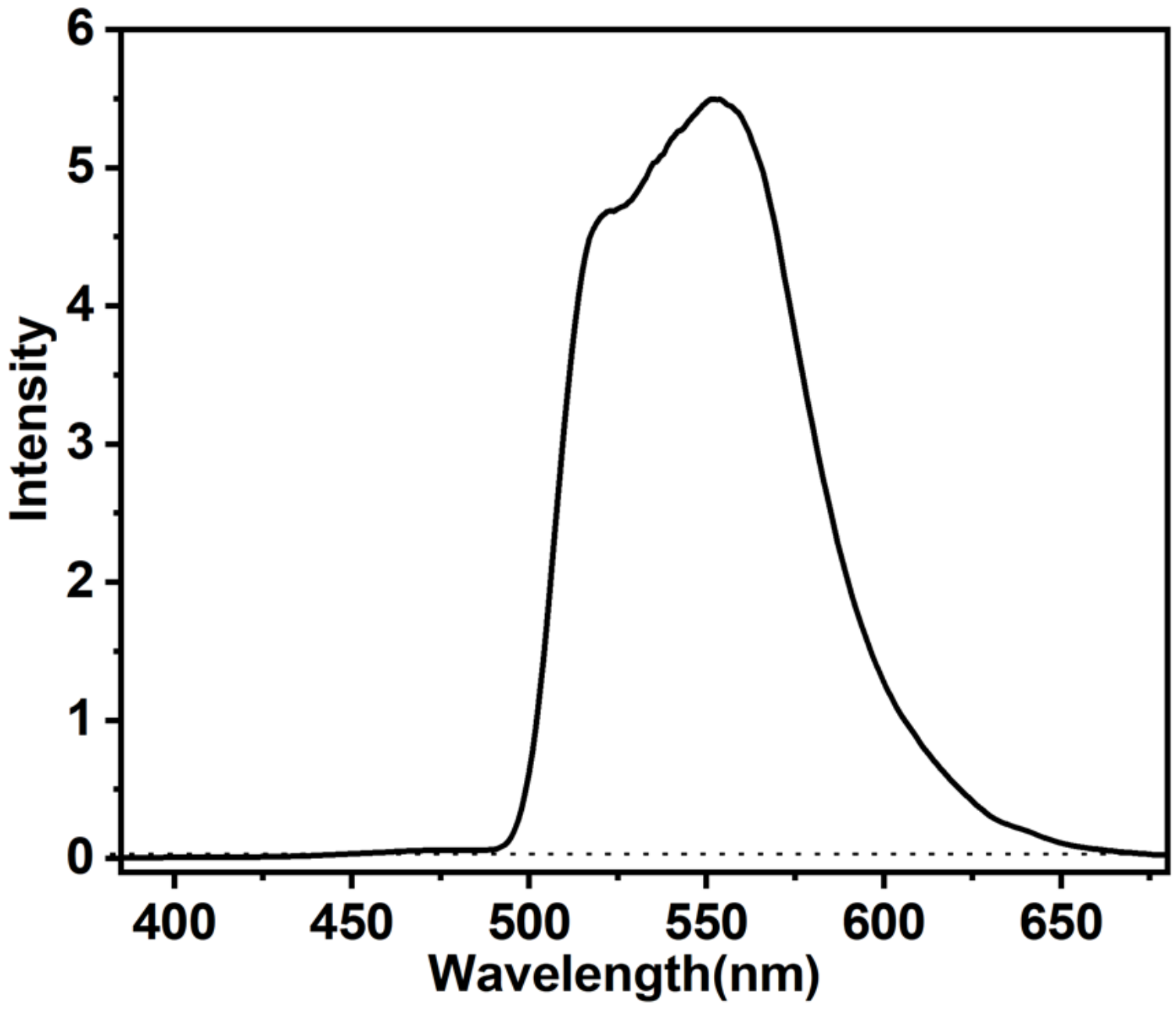

Figure S14. Phosphorescence spectra (delay 1 ms) of **TRZ-OPS** at 77K (λ_{ex} = 318 nm).

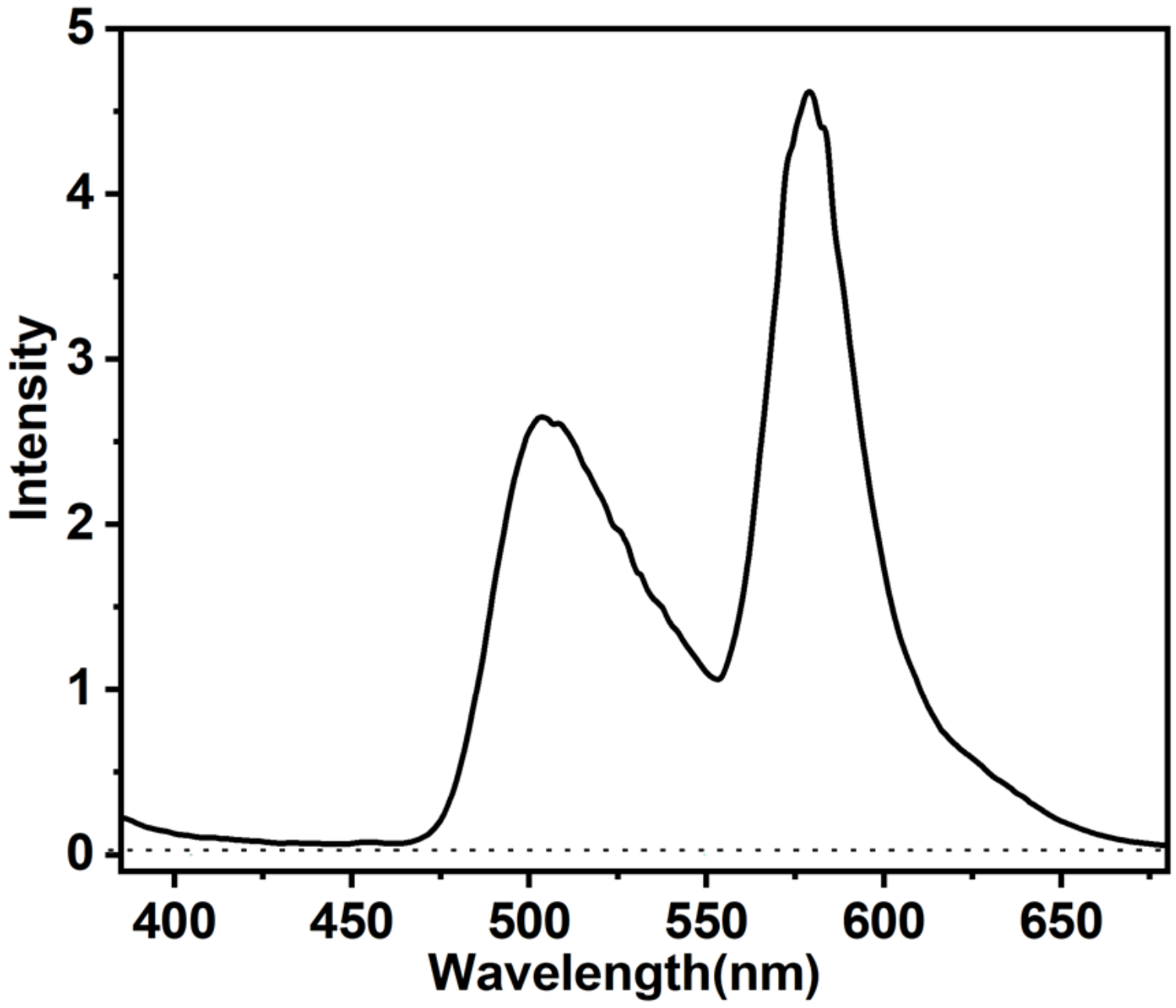

Figure S15. Steady-state PL spectra of **TRZ-TFA** at 77K (λ_{ex} = 318 nm).

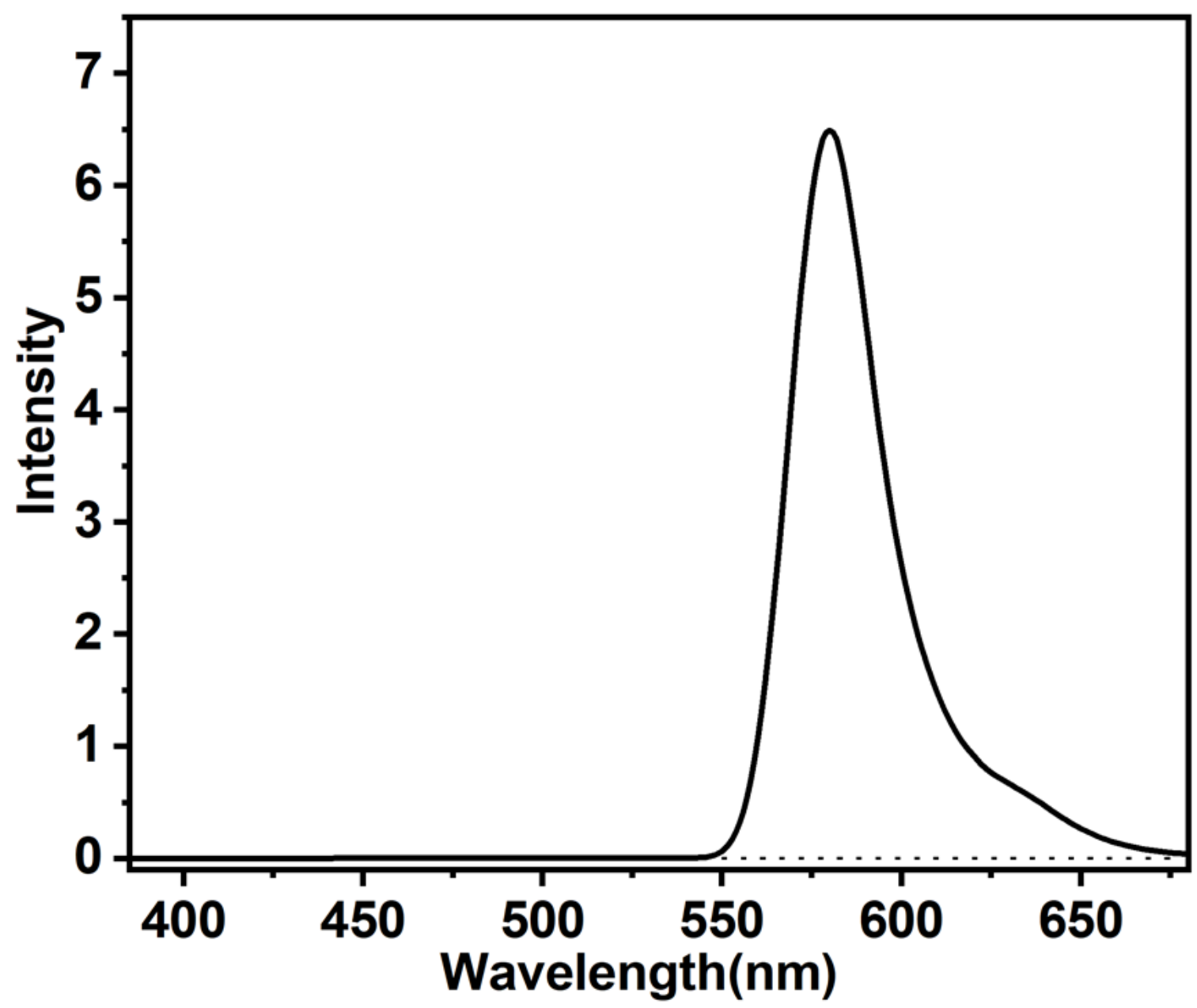

Figure S16. Phosphorescence spectra (delay 1 ms) of **TRZ-TFA** at 77K (λ_{ex} = 318 nm).

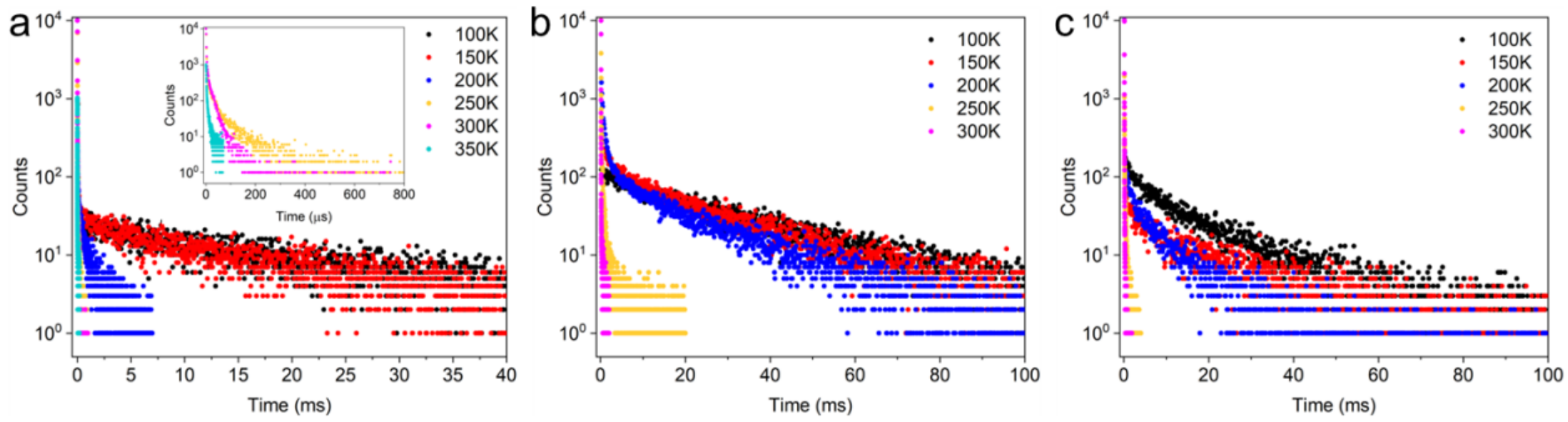

Figure S17. Temperature-dependent lifetime decay curves of (a) TRZ-ESA, (b) TRZ-TFA, and (c) TRZ-OPS.

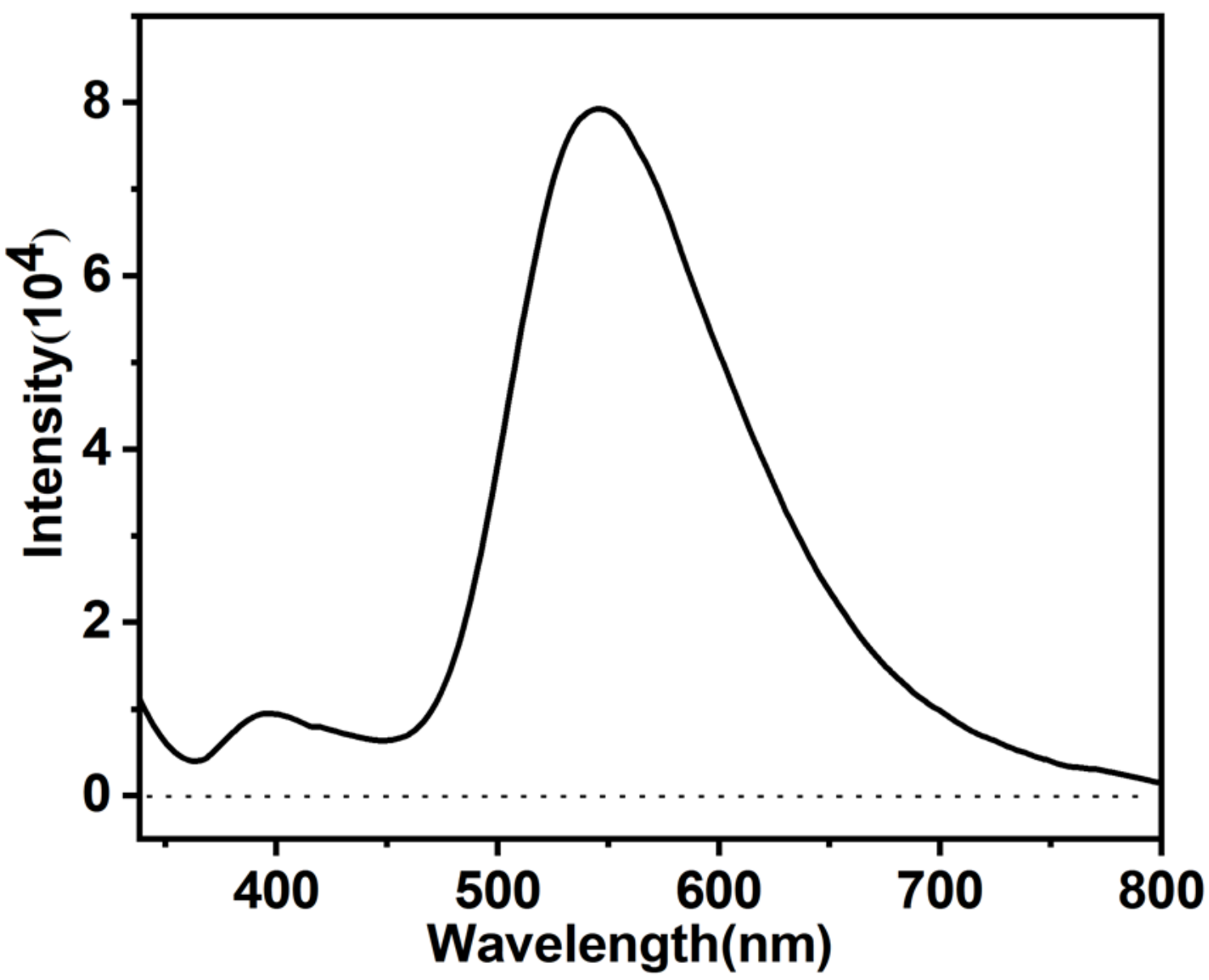

Figure S18. Emission spectra of annealed **TRZ-OPS** (λ_{ex} = 318 nm).

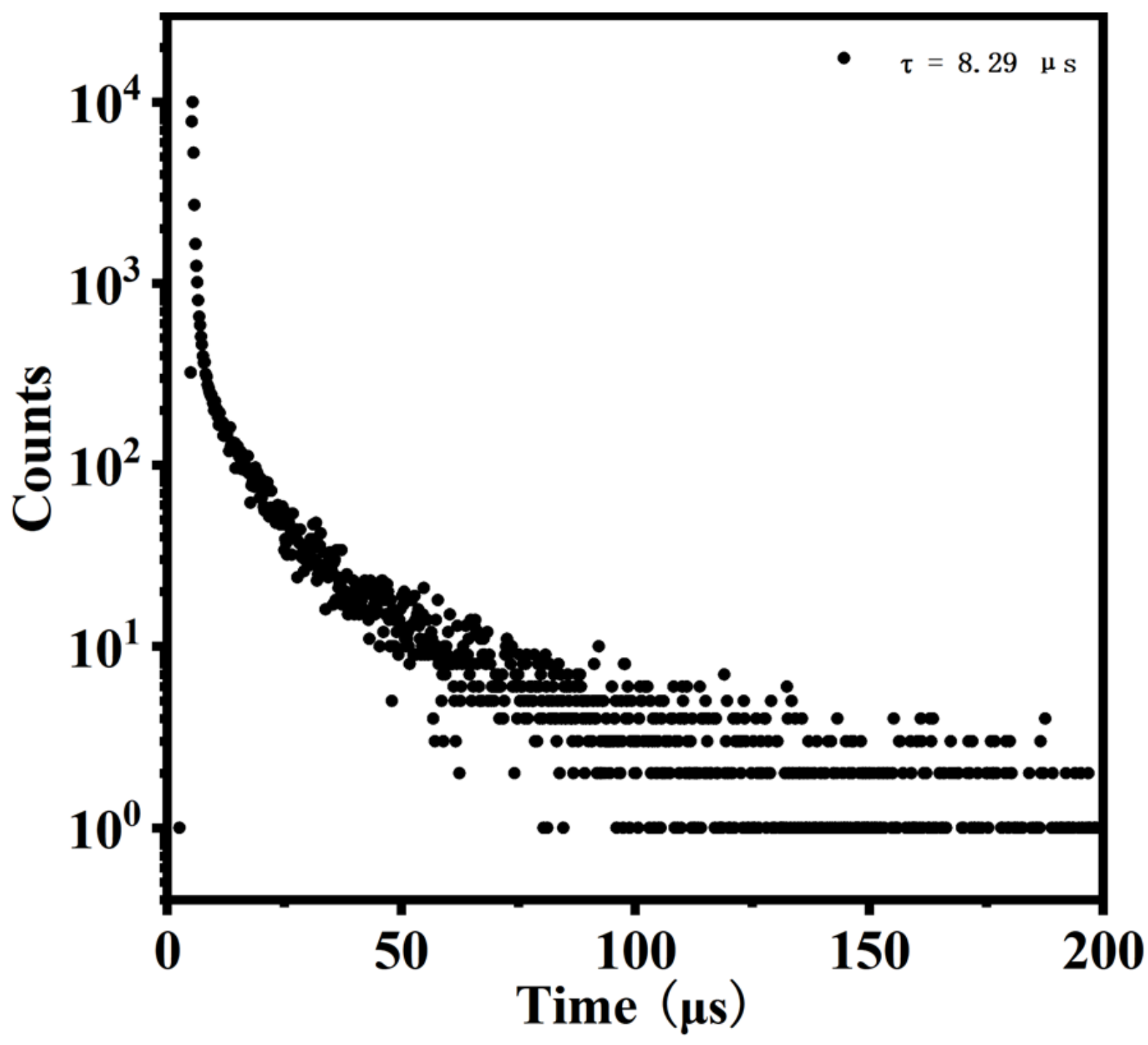

Figure S19. Time-resolved decay curves of annealed **TRZ-OPS** (λ_{ex} = 318 nm, λ_{em} = 550 nm)

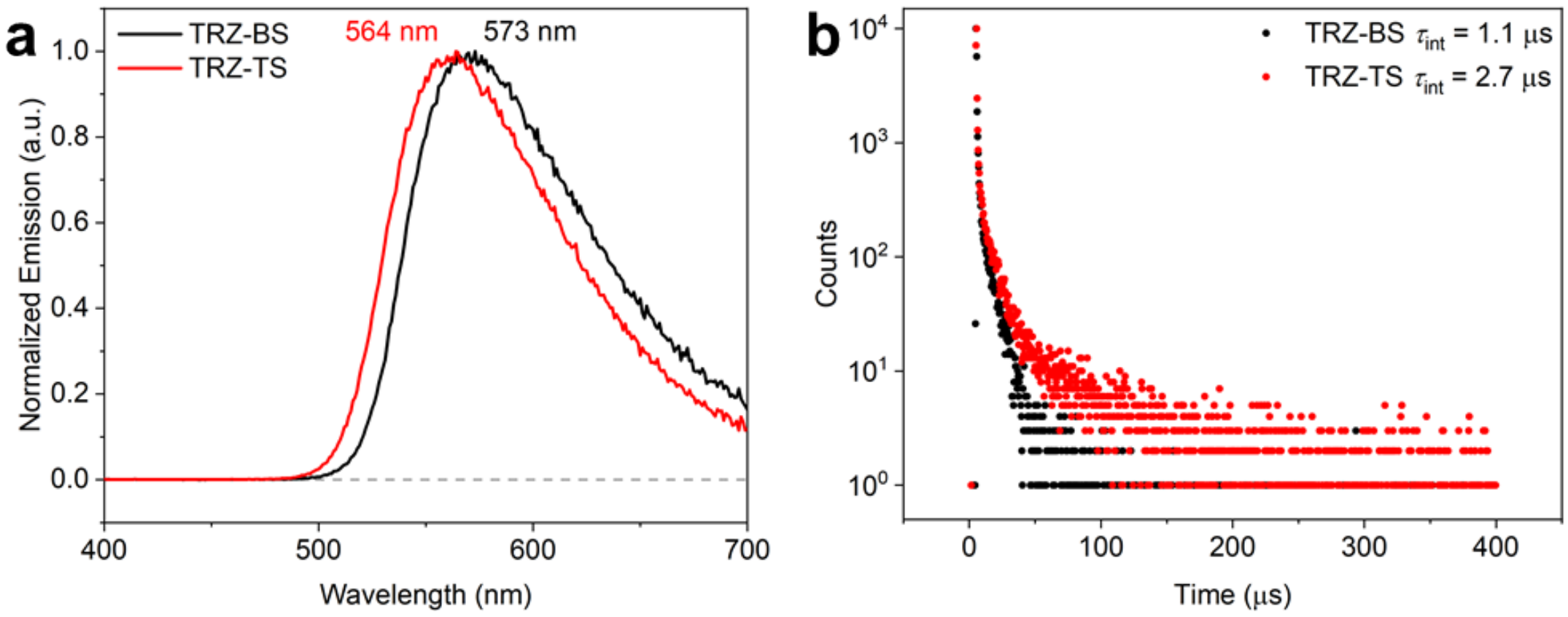

Figure S20. Photophysical properties of TRZ-BS and TRZ-TS. (a) Normalized emission and (b) lifetime decay curves.

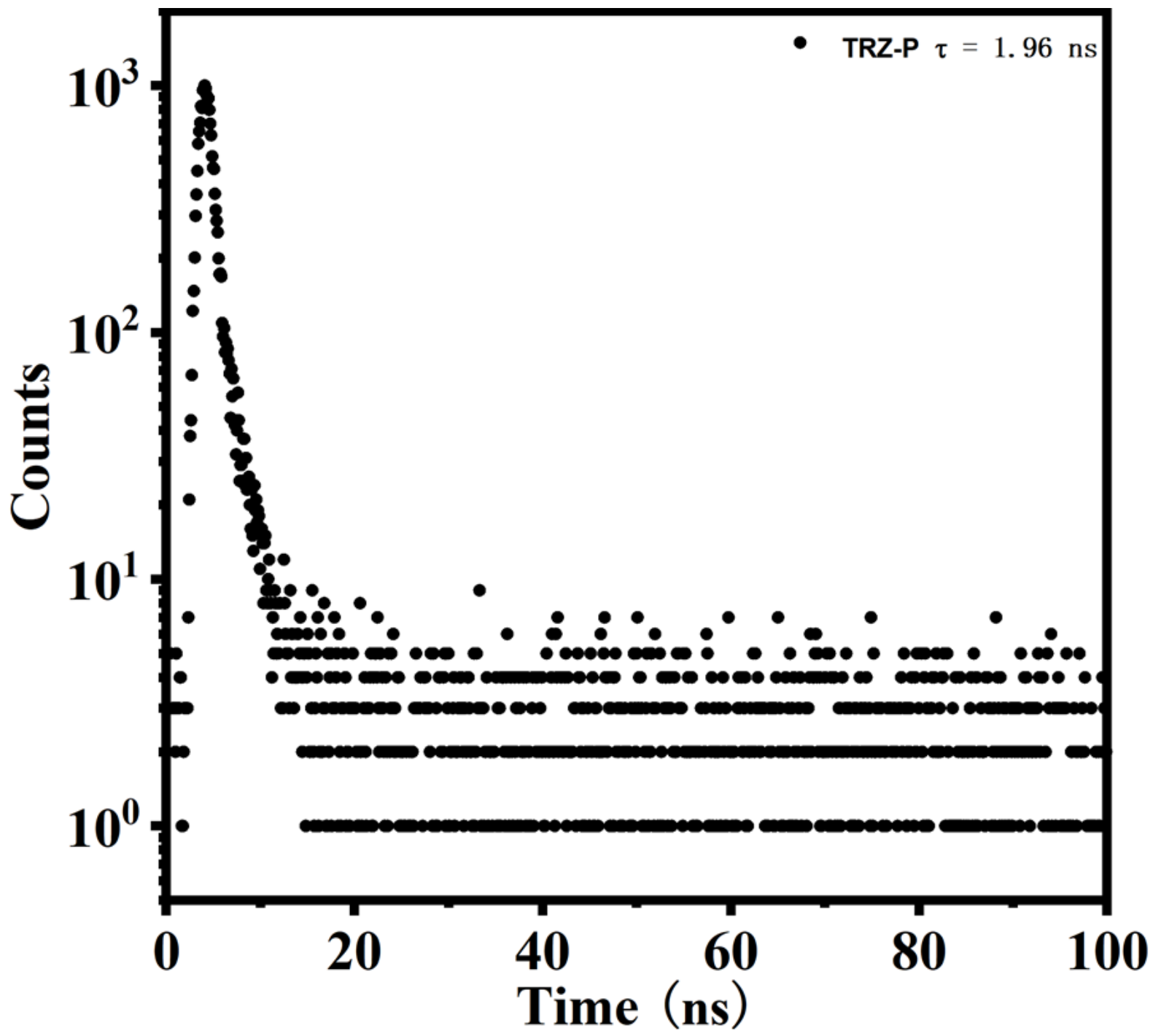

Figure S21. Time-resolved decay curves of annealed **TRZ-P** (λ_{ex} = 310 nm, λ_{em} = 400 nm).

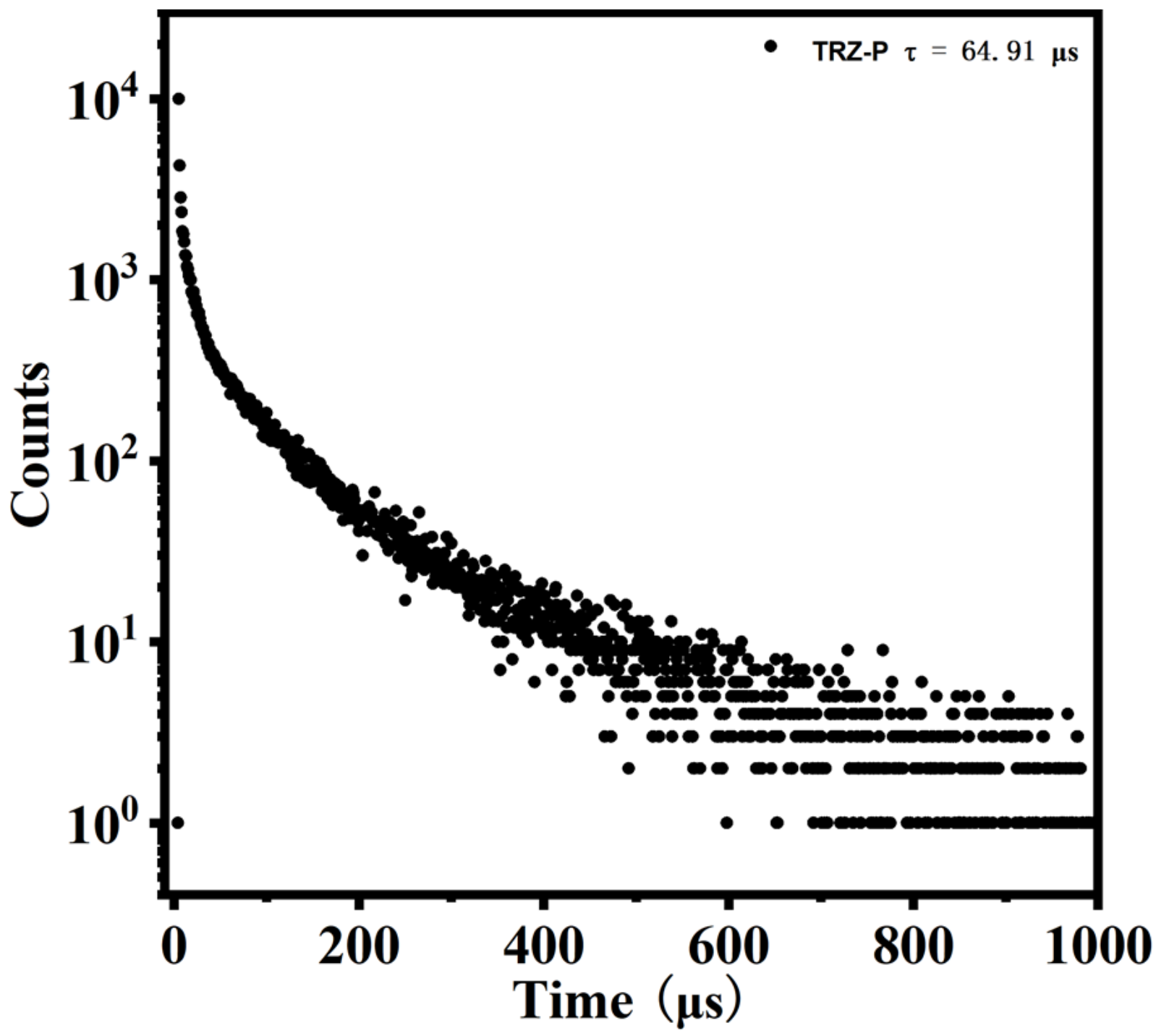

Figure S22. Time-resolved decay curves of annealed **TRZ-P** (λ_{ex} = 318 nm, λ_{em} = 550 nm).

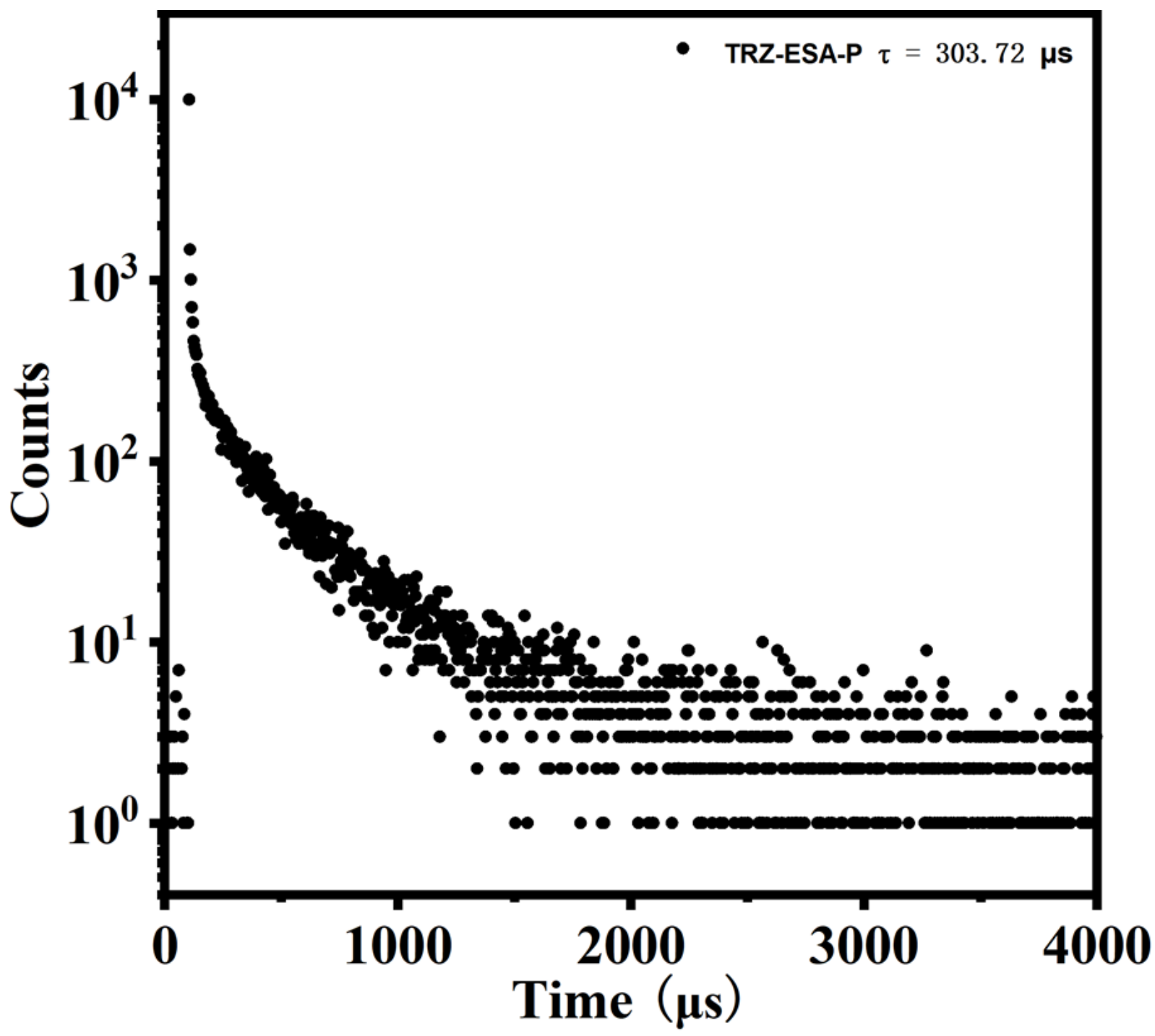

Figure S23. Time-resolved decay curves of annealed **TRZ-ESA-P** (λ_{ex} = 318 nm, λ_{em} = 550 nm).

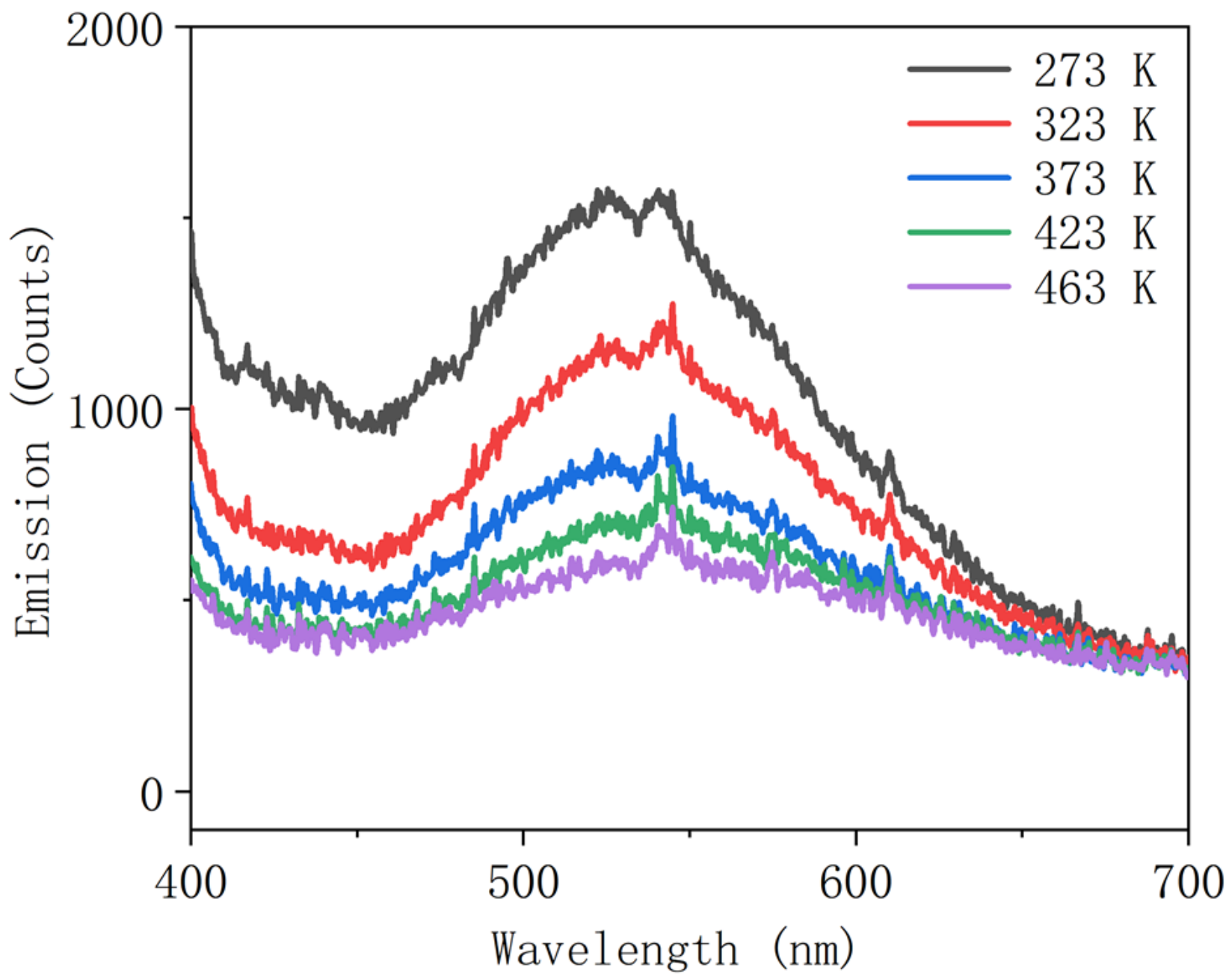

Figure S24. Temperature-dependent emission of **TRZ-OPS** under 370 nm excitation.

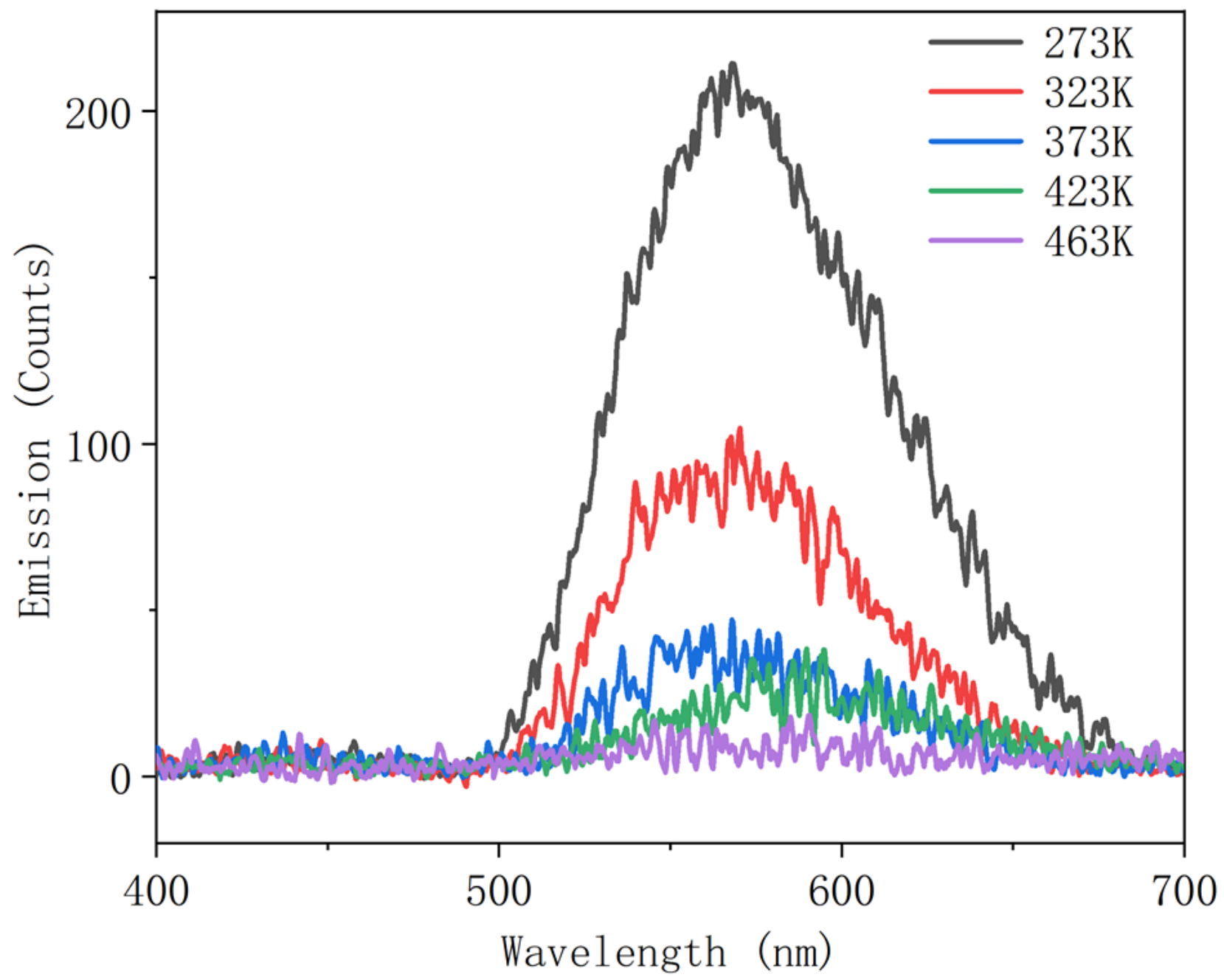

Figure S25. Temperature-dependent emission of **TRZ-TFA** under 370 nm excitation.

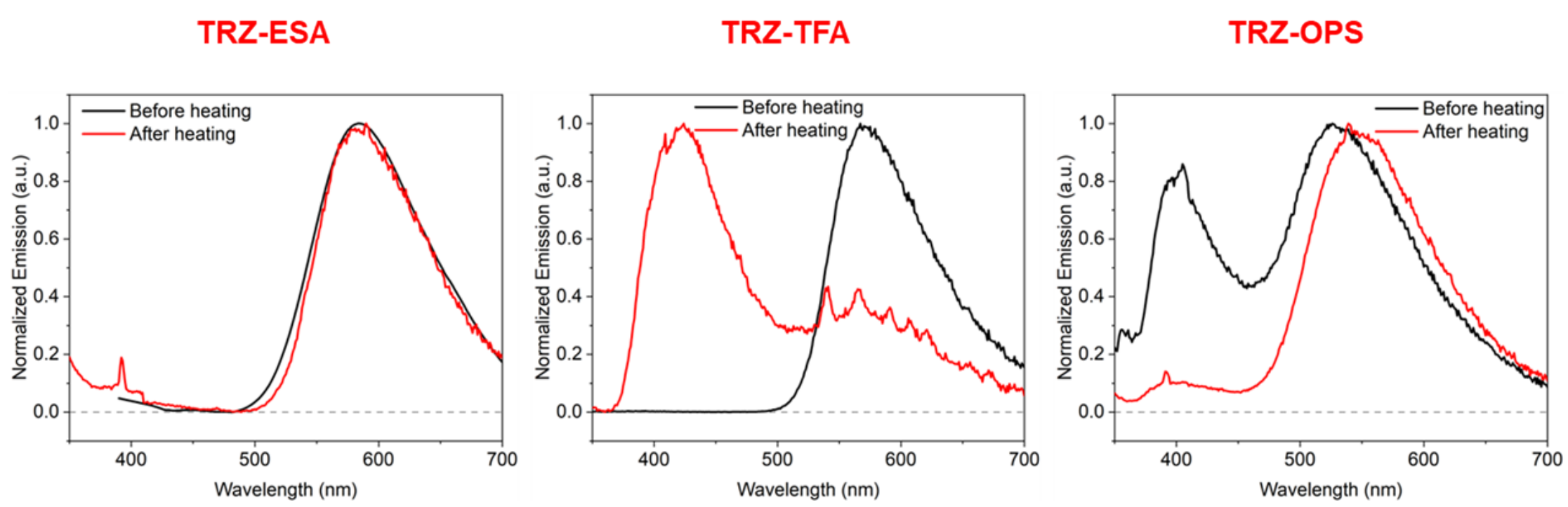

Figure S26. Comparison of powder samples at room temperature before and after heating.

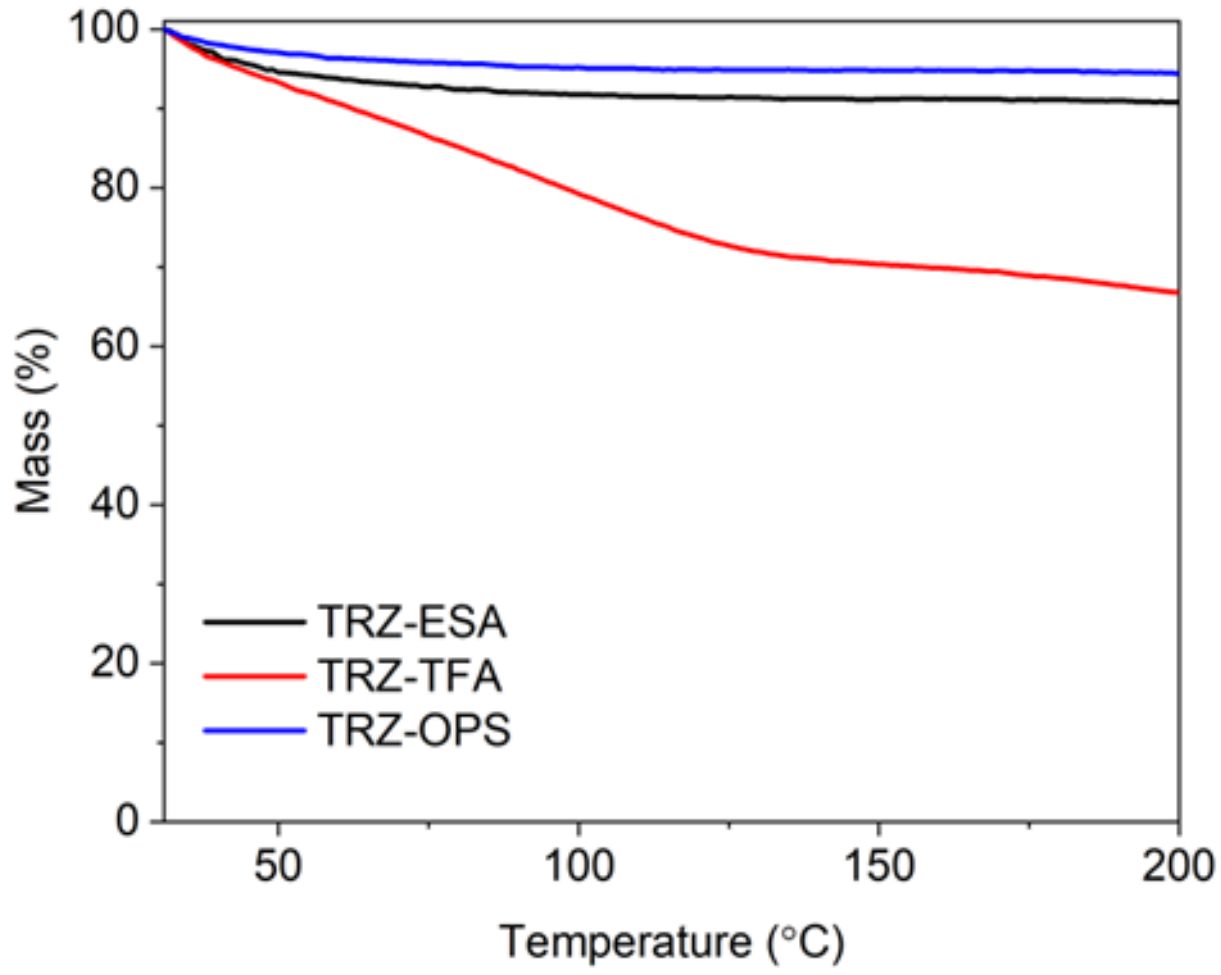

Figure S27. TGA curves of ground samples.

Table S1. Temperature-dependent lifetime decays.

Sample	τ_1 (τ_1%)	τ_2 (τ_2%)	τ_3 (τ_3%)	χ^2	τ_{int}
TRZ-ESA (100 K)	2680 μs (4.2%)	23388 μs (95.8%)	-	1.1378	22510 μs
TRZ-ESA (150 K)	853 μs (4.9%)	14555 μs (95.1%)	-	1.1561	13882 μs
TRZ-ESA (200 K)	23.1 μs (29.6%)	513 μs (70.4%)	-	1.0566	369 μs
TRZ-ESA (250 K)	1.33 μs (35.3%)	14.3 μs (35.0%)	71.7 μs (29.7%)	1.0993	26.8 μs
TRZ-ESA (300 K)	0.80 μs (46.5%)	3.79 μs (20.8%)	19.3 μs (33.0%)	1.3079	7.53 μs
TRZ-ESA (350 K)	1.42 μs (63.2%)	8.72 μs (36.7%)	-	1.3916	4.10 μs
TRZ-TFA (100 K)	10099 μs (10.8%)	34818 μs (89.2%)	-	1.0913	32142 μs
TRZ-TFA (150 K)	2574 μs (11.2%)	22542 μs (88.8%)	-	1.1524	20310 μs
TRZ-TFA (200 K)	800 μs (24.7%)	17196 μs (75.3%)	-	1.1416	13151 μs
TRZ-TFA (250 K)	26.8 μs (32.2%)	202 μs (51.7%)	933 μs (16.0%)	0.9540	263 μs
TRZ-TFA (300 K)	2.01 μs (95.7%)	164 μs (4.3%)	-	1.2243	8.89 μs
TRZ-OPS (100 K)	2204 μs (12.1%)	15460 μs (87.9%)	-	1.1239	13853 μs

TRZ-OPS (150 K)	1666 μs (12.1%)	18351 μs (87.9%)	-	1.1345	16338 μs
TRZ-OPS (200 K)	872 μs (18.4%)	7017 μs (81.6%)	-	1.0333	5887 μs
TRZ-OPS (250 K)	22.3 μs (45.0%)	182 μs (55.0%)	-	1.0484	110 μs
TRZ-OPS (300 K)	4.08 μs (88.2%)	43.2% (11.8%)	-	1.1288	8.70 μs

Table S2. Details of τ values.

Sample	τ_1 (τ_1%)	τ_2 (τ_2%)	τ_3 (τ_3%)	χ^2	τ_{int}
TRZ-OPS (as prepared)	0.32 μs (89.9%)	5.38 μs (2.7%)	26.7 μs (7.4%)	1.1778	2.40 μs
TRZ-OPS (after annealing)	0.35 μs (39.8%)	4.35 μs (29.9%)	22.6 μs (30.3%)	1.1573	8.29 μs
TRZ-TFA	0.40 μs (83.9%)	30.0 μs (16.1%)	-	1.2126	5.17 μs
TRZ-ESA	0.80 μs (46.2%)	3.79 μs (20.8%)	19.3 μs (33.0%)	1.3079	7.53 μs
TRZ-ESA (100 μs of gating)	9.88 μs (21.1%)	42.3 μs (38.1%)	164 μs (40.8%)	1.1358	85.2 μs

Table S3. Comparison table of organic small molecular HTP materials.

Material	λ_{phos} (nm)	τ_{phos}	$PLQY_{phos}$	T_{max} (°C)	Strategy	Reference
**TRZ-ESA**	**585**	**7.53 μs**	**8.3%**	**190**	**Hydrogen bonding**	**This work**
**TRZ-TFA**	**563**	**5.17 μs**	**14%**	**100**		
**TRZ-OPS**	**528**	**2.40 μs**	**1.5%**	**190**		
1NaP/BTA	528	653 ms	61%	187	Host-Guest doping	*Angew. Chem. Int. Ed.* **2026**, *65*, e4158258
CoTAK@BAK	535, 580, 623	2.7 s	38.7%	100	Host-Guest doping	*J. Am. Chem. Soc.* **2025**, *147*, 11098-11107
TFH/BTA	522	3.8 s	56.8%	200	Host-Guest doping	*Nat. Commun.* **2026**, *17*, 1870
TCPB@BO	530	1805 ms	20.56%	230	Host-Guest doping	*Chem. Eng. J.* **2025**, *516*, 164264
MCTPHA	510	893 ms	no report	203	Multivalent	*Angew.*

					assembly	*Chem. Int. Ed.* **2025**, *64*, e202423650
COR-MF	568	4.83 s	22.7%	167	Covalent confinement	*Angew. Chem. Int. Ed.* **2024**, *63*, e202318516
CC1	477	2.16 s	4.5%	150	Cocrystal engineering	*Angew. Chem. Int. Ed.* **2024**, *63*, e202319694

4. DFT calculations

Geometry optimizations were performed at the B3LYP/6-311G(d,p) level with subsequent frequency calculations to confirm that local minima were obtained. The electronic excitation properties were further investigated based on the time-dependent density functional theory (TD-DFT) method. All calculations were conducted using the Gaussian 16 C.01 program package.[1] The electric and magnetic transition dipole moments of were calculated by Multiwfn.[2]

Cartesian coordinates from calculation of TRZ in excited state:

Center Number	Atomic Number	Atomic Type	Coordinates (Angstroms) X	Y	Z
1	7	0	0.369845	-1.320976	0.008845
2	6	0	-0.917125	-0.939213	0.010509
3	7	0	-1.324186	0.339886	0.006422
4	6	0	-0.350185	1.263585	0.000705
5	7	0	0.961064	0.976740	-0.000170
6	6	0	1.273837	-0.328788	0.003760
7	6	0	2.704359	-0.696803	0.002426
8	6	0	-0.747367	2.686240	-0.004510
9	6	0	-1.951454	-1.993803	0.015812
10	6	0	-3.313315	-1.656668	0.013904
11	6	0	-4.284795	-2.648904	0.016792
12	6	0	-3.929820	-4.009160	0.023066
13	6	0	-2.563096	-4.338725	0.026413
14	6	0	-1.588931	-3.349204	0.022041
15	6	0	0.223807	3.698576	-0.019457
16	6	0	-0.152432	5.035385	-0.025987
17	6	0	-1.508538	5.405453	-0.015758
18	6	0	-2.475582	4.385108	0.000769
19	6	0	-2.103158	3.047481	0.005158
20	6	0	3.695947	0.295687	-0.004159

21	6	0	5.040246	-0.052056	-0.006077
22	6	0	5.439233	-1.400141	0.000488
23	6	0	4.439712	-2.388561	0.007049
24	6	0	3.094329	-2.044521	0.007398
25	6	0	6.872752	-1.766787	-0.000722
26	6	0	-1.907801	6.829964	-0.022206
27	6	0	-4.965660	-5.065573	0.025841
28	6	0	-4.763690	-6.274667	-0.659814
29	6	0	-5.733698	-7.273600	-0.663197
30	6	0	-6.923132	-7.062245	0.029880
31	6	0	-7.156787	-5.875796	0.720941
32	6	0	-6.177283	-4.886245	0.713394
33	6	0	-1.146395	7.795370	0.656678
34	6	0	-1.515485	9.137975	0.655450
35	6	0	-2.662003	9.522417	-0.035609
36	6	0	-3.440211	8.591977	-0.720091
37	6	0	-3.058142	7.253036	-0.708167
38	35	0	-8.262614	-8.428905	0.032658
39	35	0	-3.178105	11.365067	-0.044991
40	6	0	7.326130	-2.905718	-0.686040
41	6	0	8.673771	-3.256112	-0.692350
42	6	0	9.582337	-2.457593	-0.002016
43	6	0	9.167568	-1.322015	0.689613
44	6	0	7.816696	-0.984615	0.685016
45	35	0	11.436730	-2.929756	-0.003170
46	1	0	-3.595220	-0.610955	0.000283
47	1	0	-5.332204	-2.368117	-0.012230
48	1	0	-2.263499	-5.380856	0.058243
49	1	0	-0.538167	-3.611883	0.032549
50	1	0	1.270925	3.421959	-0.035589

51	1	0	0.612499	5.803610	-0.065760
52	1	0	-3.528081	4.645296	0.036824
53	1	0	-2.853768	2.266803	0.025870
54	1	0	3.396637	1.336545	-0.017363
55	1	0	5.792312	0.728971	-0.039284
56	1	0	4.721053	-3.435806	0.038118
57	1	0	2.330057	-2.811992	0.021084
58	1	0	-3.847977	-6.429815	-1.220195
59	1	0	-5.570595	-8.198491	-1.203462
60	1	0	-8.083611	-5.729712	1.262773
61	1	0	-6.349514	-3.972604	1.271958
62	1	0	-0.267643	7.492600	1.215802
63	1	0	-0.925343	9.872248	1.190697
64	1	0	-4.324572	8.907929	-1.260455
65	1	0	-3.650409	6.532910	-1.262479
66	1	0	6.622867	-3.514347	-1.244110
67	1	0	9.012807	-4.131762	-1.232924
68	1	0	9.885152	-0.716151	1.229897
69	1	0	7.491006	-0.114573	1.244987

Cartesian coordinates from calculation of TRZ-ESA in excited state:

Center Number	Atomic Number	Atomic Type	Coordinates (Angstroms) X	Y	Z
1	7	0	0.136002	0.860600	-0.043045
2	6	0	-0.110969	-0.444246	-0.402437
3	7	0	0.870890	-1.315490	-0.385173
4	6	0	2.129318	-0.895218	-0.263745

5	7	0	2.397152	0.417902	-0.229712
6	6	0	1.430280	1.298767	-0.198280
7	6	0	1.715068	2.689827	-0.267481
8	6	0	3.194974	-1.839800	-0.249607
9	6	0	-1.426619	-0.838587	-0.749538
10	6	0	-1.811140	-2.170431	-0.931847
11	6	0	-3.117256	-2.526955	-1.185262
12	6	0	-4.161038	-1.603516	-1.323513
13	6	0	-3.743326	-0.273131	-1.245441
14	6	0	-2.447834	0.092758	-0.962100
15	6	0	4.531491	-1.459635	-0.161172
16	6	0	5.556419	-2.377133	-0.145264
17	6	0	5.355515	-3.755882	-0.214038
18	6	0	4.011750	-4.121376	-0.300480
19	6	0	2.978802	-3.213742	-0.317174
20	6	0	3.009332	3.190004	-0.383866
21	6	0	3.270199	4.537332	-0.445445
22	6	0	2.275998	5.517469	-0.400182
23	6	0	0.985612	5.002240	-0.289522
24	6	0	0.712314	3.655070	-0.226987
25	6	0	2.556532	6.921033	-0.463250
26	6	0	6.427027	-4.706892	-0.196438

27	6	0	-5.527739	-1.982815	-1.513302
28	6	0	-6.555670	-1.051553	-1.670529
29	6	0	-7.878916	-1.403204	-1.816903
30	6	0	-8.242782	-2.731901	-1.816186
31	6	0	-7.264587	-3.690602	-1.671036
32	6	0	-5.947287	-3.314507	-1.525240
33	6	0	6.216455	-6.084610	-0.265184
34	6	0	7.237617	-7.009039	-0.249004
35	6	0	8.545822	-6.588463	-0.161342
36	6	0	8.807351	-5.238339	-0.091029
37	6	0	7.768670	-4.333605	-0.109093
38	35	0	-10.066166	-3.234335	-2.006109
39	35	0	9.967650	-7.851056	-0.138048
40	6	0	1.558416	7.894553	-0.416669
41	6	0	1.816045	9.246191	-0.476228
42	6	0	3.113096	9.695400	-0.587354
43	6	0	4.135147	8.773972	-0.636972
44	6	0	3.851520	7.427456	-0.575562
45	35	0	3.486526	11.557586	-0.670699
46	6	0	-0.123072	0.900774	1.504029
47	6	0	-1.518893	1.411660	1.910282
48	16	0	-2.361298	0.278658	3.082576

49	8	0	-1.485055	0.384607	4.271370
50	8	0	-4.049834	1.093238	3.118695
51	8	0	-2.359163	-1.061003	2.256987
52	6	0	-7.621771	-2.169145	2.313923
53	6	0	-6.121003	-2.305452	2.048826
54	16	0	-5.305658	-0.728448	1.889061
55	8	0	-5.794427	0.056919	0.743670
56	8	0	-3.548566	-1.123082	1.419195
57	8	0	-5.069555	0.023546	3.220895
58	1	0	-4.794969	1.864832	3.133692
59	1	0	-3.018021	-1.451508	0.572821
60	1	0	-1.083683	-2.978673	-0.817790
61	1	0	-3.284816	-3.604261	-1.253132
62	1	0	-4.432262	0.563971	-1.372393
63	1	0	-2.231549	1.164424	-0.928357
64	1	0	4.803090	-0.401442	-0.099830
65	1	0	6.554725	-1.938104	-0.072658
66	1	0	3.696082	-5.166150	-0.359606
67	1	0	1.961626	-3.611452	-0.384980
68	1	0	3.865048	2.510075	-0.427429
69	1	0	4.330029	4.788263	-0.533534
70	1	0	0.103375	5.645619	-0.246318

71	1	0	-0.339158	3.362125	-0.145123
72	1	0	-6.374784	0.024835	-1.680712
73	1	0	-8.636190	-0.621104	-1.929238
74	1	0	-7.526886	-4.753148	-1.662608
75	1	0	-5.253226	-4.148591	-1.401624
76	1	0	5.218059	-6.521244	-0.336901
77	1	0	7.001411	-8.076346	-0.306285
78	1	0	9.838037	-4.876566	-0.020809
79	1	0	8.082991	-3.289477	-0.048816
80	1	0	0.499161	7.643669	-0.328837
81	1	0	0.985048	9.957261	-0.434433
82	1	0	5.175001	9.103828	-0.724993
83	1	0	4.730868	6.781689	-0.622465
84	1	0	0.625543	1.515607	2.039182
85	1	0	0.051616	-0.096459	1.951275
86	1	0	-2.175341	1.544978	1.037698
87	1	0	-1.468551	2.409562	2.379127
88	1	0	-8.079031	-3.169320	2.408748
89	1	0	-7.819965	-1.616948	3.248381
90	1	0	-8.129643	-1.645487	1.486088
91	1	0	-5.949635	-2.866120	1.110959
92	1	0	-5.633761	-2.861754	2.869550

----

5. References

1. M. J. Frisch, G. W. Trucks, H. B. Schlegel, G. E. Scuseria, M. A. Robb, J. R. Cheeseman, G. Scalmani, V. Barone, G. A. Petersson, H. Nakatsuji, X. Li, M. Caricato, A. V. Marenich, J. Bloino, B. G. Janesko, R. Gomperts, B. Mennucci, H. P. Hratchian, J. V. Ortiz, A. F. Izmaylov, J. L. Sonnenberg, D. Williams-Young, F. Ding, F. Lipparini, F. Egidi, J. Goings, B. Peng, A. Petrone, T. Henderson, D. Ranasinghe, V. G. Zakrzewski, J. Gao, N. Rega, G. Zheng, W. Liang, M. Hada, M. Ehara, K. Toyota, R. Fukuda, J. Hasegawa, M. Ishida, T. Nakajima, Y. Honda, O. Kitao, H. Nakai, T. Vreven, K. Throssell, J. A. Montgomery Jr., J. E. Peralta, F. Ogliaro, M. J. Bearpark, J. J. Heyd, E. N. Brothers, K. N. Kudin, V. N. Staroverov, T. A. Keith, R. Kobayashi, J. Normand, K. Raghavachari, A. P. Rendell, J. C. Burant, S. S. Iyengar, J. Tomasi, M. Cossi, J. M. Millam, M. Klene, C. Adamo, R. Cammi, J. W. Ochterski, R. L. Martin, K. Morokuma, O. Farkas, J. B. Foresman and D. J. Fox, *Gaussian 16, Revision A.03*, Gaussian, Inc., Wallingford CT, 2016.
2. F. Neese, *WIREs Comput. Mol. Sci.*, 2011, **2**, 73-78.
